# Are diversification rates and chromosome evolution in the temperate grasses (Pooideae) associated with major environmental changes in the Oligocene-Miocene?

**DOI:** 10.7717/peerj.3815

**Published:** 2017-09-22

**Authors:** Manuel Pimentel, Marcial Escudero, Elvira Sahuquillo, Miguel Ángel Minaya, Pilar Catalán

**Affiliations:** 1 Evolutionary Biology Research Group (GIBE), Department of Biology, University of A Coruña, A Coruña, Galicia, Spain; 2 Department of Plant Biology and Ecology, University of Sevilla, Sevilla, Andalucía, Spain; 3 Department of Molecular Microbiology and Immunology, St. Louis University, Saint Louis, MO, United States of America; 4 High Polytechnic School of Huesca, University of Zaragoza, Huesca, Aragón, Spain; 5 Department of Botany, Institute of Biology, Tomsk State University, Tomsk, Russia

**Keywords:** Chromosome changes, Diversification rates, Evolution, C3 grasses, Phylogeny, Polyploidy, Pooideae

## Abstract

The Pooideae are a highly diverse C3 grass subfamily that includes some of the most economically important crops, nested within the highly speciose core-pooid clade. Here, we build and explore the phylogeny of the Pooideae within a temporal framework, assessing its patterns of diversification and its chromosomal evolutionary changes in the light of past environmental transformations. We sequenced five plastid DNA loci, two coding (*ndhF*, *matk*) and three non-coding (*trnH-psbA*, *trnT-L* and *trnL-F*), in 163 Poaceae taxa, including representatives for all subfamilies of the grasses and all but four ingroup Pooideae tribes. Parsimony and Bayesian phylogenetic analyses were conducted and divergence times were inferred in BEAST using a relaxed molecular clock. Diversification rates were assessed using the MEDUSA approach, and chromosome evolution was analyzed using the chromEvol software. Diversification of the Pooideae started in the Late-Eocene and was especially intense during the Oligocene-Miocene. The background diversification rate increased significantly at the time of the origin of the Poodae + Triticodae clade. This shift in diversification occurred in a context of falling temperatures that potentially increased ecological opportunities for grasses adapted to open areas around the world. The base haploid chromosome number *n* = 7 has remained stable throughout the phylogenetic history of the core pooids and we found no link between chromosome transitions and major diversification events in the Pooideae.

## Introduction

A combination of phylogenetic inference and cytological and taxonomic diversity information is commonly used to investigate the *tempo* and *mode* of diversification and the impact of chromosome changes in diversity (e.g., Escudero et al., 2012). Here, we use such approach in order to assess the diversification of the grass subfamily Pooideae through time, as well as to analyse the impact of chromosome changes in the evolution of the subfamily.

The grass monophyletic subfamily Pooideae comprises about one third of the grasses (*ca*. 177 genera and *ca*. 3850 species *sensu* Kellogg, 2015a or *ca*. 197 genera and *ca*. 4234 species *sensu* Soreng et al., 2015, including some of the most prominent crops such as wheat, rye, oats and barley (Clayton & Renvoize, 1986; GPWG, 2001; Kellogg, 2015a). Its phylogenetic structure has been thoroughly studied (e.g., GPWG, 2001; Bouchenak-Khelladi et al., 2008, and references therein), although different authors have called for larger datasets to increase the robustness of the results (GPWG, 2012; Soreng et al., 2015; Saarela et al., 2015).

The systematic positions of the different tribes and subtribes within the Pooideae are currently under discussion, and their evolutionary relationships are not totally resolved (e.g., Bouchenak-Khelladi et al., 2008; Pirie et al., 2008; Sanchez-Ken & Clarke, 2010; Cahoon et al., 2010; GPWG, 2012; Soreng et al., 2015; Kellogg, 2015a). The tribal arrangement of the Pooideae has varied widely over the last century (e.g., Ascherson & Graebner, 1898–1902; Clayton & Renvoize, 1986; Tzvelev, 1989; Watson & Dallwitz, 1992; Kellogg, 2015a; Soreng et al., 2015) and thirteen tribes have been long recognised in the subfamily based on combined molecular and morphological data (Hsiao et al., 1995; Catalán, Kellogg & Olmstead, 1997; GPWG, 2001). Recent works with broader sampling have uncovered inconsistencies in this taxonomic arrangement (Gillespie, Archambault & Soreng, 2007; Quintanar, Castroviejo & Catalán, 2007; Catalán et al., 2007; Döring et al., 2007; Soreng et al., 2007; Soreng et al., 2015; Saarela et al., 2010) and a wide range of taxonomic changes have been suggested (Jacobs et al., 2007; Schneider et al., 2009; Soreng et al., 2015). The Aveneae and Poeae tribes were merged into a supertribe Poodae (including tribes Hainardeae, Phleeae and Seslerieae (Macfarlane & Watson, 1982; Macfarlane, 1987; Watson & Dallwitz, 1992) or tribe Poeae *s.l.* (Davis & Soreng, 2007; Soreng et al., 2003; Soreng et al., 2007; Quintanar, Castroviejo & Catalán, 2007) based on molecular phylogenetic evidence. Poodae has been split into 19 or 21 subtribes, arranged into two groups based on plastid DNA (Fig. S1). In the most recent classification, twelve subtribes (plus the *incertae sedis Avenula*—*Homalotrichon*) belong to the Poeae-type plastid DNA clade and seven to the Aveneae-type plastid DNA clade (Soreng et al., 2015). Different studies focusing on some particular subtribes have suggested that further changes to the taxonomy of the supertribe Poodae may be necessary (e.g., Catalán, 2006; Saarela et al., 2010). A supertribe Triticodae (Macfarlane & Watson, 1982) has also been proposed including three tribes: Bromeae, Triticeae (encompassing subtribes Triticinae and Hordeinae) and the recently created Littledaleeae (Soreng et al., 2015). The sisters Poodae and Triticodae constitute the “core pooids”, a highly speciose lineage formed by taxa showing some of the largest genomes of grasses due to the accumulation of transposons (e.g., Kellogg, 2015b).

Dated grass phylogenies (most of them primarily focused in C4 grasses; Vicentini et al., 2008; Bouchenak-Khelladi et al., 2010) have placed the diversification of the BOP (Bambusoideae, Oryzoideae and Pooideae, *sensu* Soreng et al., 2015) clade between the Late Paleocene and the Early Oligocene (Vicentini et al., 2008), although dates are heavily dependent on the choice of fossils for calibration (e.g., Christin et al., 2008; Strömberg, 2011). Diversification within the Pooideae took place mostly during the Oligocene and in the Neogene, in a process that paralleled the expansion of grasslands (Edwards et al., 2010; Spriggs, Christin & Edwards, 2014), although whether both processes were coupled is still an open question (Strömberg, 2005). Deep climatic transformations accompanied the diversification and ecological expansion of the Pooideae (Edwards et al., 2010), although this process differed widely across regions (Strömberg, 2005). Generally speaking, drought-tolerant grasses were already present in the Eocene, and they became common in fossil assemblages from the Miocene (Strömberg, 2005). This process is coincident with the Neogene (and the Oligocene) climatic deterioration, characterised by a fall in temperatures and CO_2_ atmospheric concentration (Retallack, 2001).

Different methods have been used to measure diversification rates in angiosperms, producing contrasting results (e.g., Magallón & Sanderson, 2001; Magallón & Castillo, 2009). Rates in the Poales have been estimated to be between *r* = 0.0611 spp. Myr^−1^ and *r* = 0.1013 spp. Myr^−1^, depending on the parameters of the analysis (Magallón & Sanderson, 2001; Magallón & Castillo, 2009); however, no information on rates is available for the Pooideae. Regarding chromosome base numbers in the subfamily, *x* = 7 is considered to be the most common base number in most groups (e.g., Triticeae, Salse et al., 2008; Poodae, Hsiao et al., 1995). A decreasing aneuploid series was proposed for this subfamily (Catalán, Kellogg & Olmstead, 1997), ranging from *x* = 13, *x* = 10 in the earlier splitting Lygeeae (and Brachyelytreae, *x* = 11; Saarela et al., 2003), through *x* = 12, 11, 10, 9, 8 in the successively diverged Stipeae, Meliceae and Brachypodieae, to *x* = 7 in the more recently split core pooids (Poodae + Triticodae, including the former tribes Poeae and Aveneae and Triticeae and Bromeae). A reduction to very small chromosome base numbers has also been documented in some pooid lineages (e.g., *x* = 5 Brachypodieae (Catalán & Olmstead, 2000); *x* = 5 Anthoxanthinae (Pimentel et al., 2013); *x* = 2 *Zingeria* P.A.Smirn. and *Colpodium* Trin. (Kim et al., 2009)). Polyploidization is common in many Pooideae and it is often linked to reticulate evolution (e.g., Catalán, 2006). The effects of chromosome mutations (including polyploidization) in the diversification of plants are controversial, and two main different views have been put forward: polyploids as evolutionary “*dead-ends*” (e.g., Mayrose et al., 2011) and polyploidy as a major force of plant evolution (e.g., Soltis et al., 2009). A third view suggests that the relationship has not been properly tested yet (Kellogg, 2016).

We have analyzed five plastid DNA loci in order to build a robust phylogenetic analysis of the Pooideae (77% of *GPWG’s (2001)* or 71,4% of Soreng et al. (2015) tribes represented in our survey; Appendix S1). Phylogenetic data, together with diversity and cytogenetic information allowed us to assess diversification through time in this group, as well as to infer trends of chromosome number evolution. We expected an increase in net diversification rates in the most speciose groups within the Pooideae, as well as a correlation between diversification in the core Pooideae and the Neogene climatic deterioration (cf. Strömberg, 2005; Vicentini et al., 2008). A relationship between diversification shifts and chromosome changes was also expected, although the effect of chromosome mutations on diversification is controversial (e.g., Soltis et al., 2014). More specifically, our aims were to: (i) analyze the diversification dynamics of the Pooideae, including the timing of divergence and the diversification rates of its main lineages; (ii) investigate the patterns of evolution of chromosomal changes operating in the core pooids, and (iii) assess the possible correlation between times of divergence, diversification rates and patterns of chromosome evolution of the main Pooideae lineages with major climatic and biome changes that occurred on Earth throughout the Cenozoic.

## Materials and Methods

### Sampling, DNA sequencing

A total of 163 species (85 genera, 61 in Pooideae) representing nine subfamilies within the Poaceae (GPWG, 2001) were included in this study. Sample details, including source, Genbank accession number and voucher information are included in Appendix S1. Sampling focused mainly on subfamily Pooideae and all currently accepted tribes (Soreng et al., 2015) except Littledaleeae, Ampelodesmeae, Phaenospermateae and Brylkinieae (realigned within Meliceae, (Schneider, 2013) or separated from it (Soreng et al., 2015)) were included in the survey. Representatives from other BOP groups and from several PACMAD (Duvall et al., 2007) lineages were added to increase the robustness of the Pooideae tree (Appendix S1). DNA sequences from five plastid DNA (cpDNA) regions, two coding (*matK*, *ndhF*) and three non-coding spacers (*trnT-L*, *trnL-F*, *trnH-psbA*) loci, were used in the phylogenetic analysis. Procedures for DNA isolation, amplification and sequencing and for sequence alignment are indicated in Appendix S2.

### Phylogenetic analyses

Separate cpDNA matrices were built for the different regions studied and phylogenetic analyses were conducted on individual and concatenated matrices. Concatenation was carried out since no conflicting node was supported by more than 0.95 Bayesian posterior probability support (PPS) or 90% bootstrap support (BS) (Pirie et al., 2009). Three different data sets were used: (i) coding regions; (ii) non-coding regions and (iii) complete data set. A two-fold analysis was applied to coding regions: (i) all sites were considered equally and (ii) non-synonymous and synonymous sites were independently analyzed. Missing sequences caused by PCR and/or sequencing failures were coded as missing data in the concatenated data sets, making a final data set with at least three sequenced genes per sample (five loci, 70.6%; four loci, 25.3%, three loci 4.1%; Appendix S1, Table S1). Overall, 798 sequences (419 newly generated sequences, and 379 sequences downloaded from GenBank, more than 60% of which were generated in projects participated by the authors) were used in this study (154 *trnH*-*psbA*; 166 *trnL-F*; 161 *trnT-L*; 149 *matK* and 168 *ndhF*; Table S1). The matrices including only coding and only non-coding regions were composed of 481 and 317 sequences, respectively.

Phylogenetic analyses were performed using maximum parsimony (MP) and Bayesian inference (BI) methods using the programs Paup v.4-10b (Swofford, 2002) and MrBayes v. 3.20 (Huelsenbeck & Ronquist, 2001), respectively. Independent analyses were conducted on each data matrix using *Joinvillea ascendens* Gaudich. ex Brongn. & Gris (Joinvillaceae) to root the tree. The MP analysis was conducted following Catalán et al. (2012), with branch support computed through 10,000 bootstrap replicates.

The GTR + I + G model was selected for all data sets using MrModelTest v2.3 (Nylander, 2004). Gaps were coded in the matrix as presence/absence following the simple method proposed by Simmons & Ochoterena (2000) as implemented in the software SeqState (Müller, 2005). All Bayesian analyses conducted with MrBayes v.3.20 were carried out with and without gaps following Ronquist, Huelsenbeck & Van der Mark (2005) and Dwivedi & Gadagkar (2009). Searches including gap characters did not improve clade support (see Results) so gaps were excluded from all final analyses.

The Bayesian analysis of each data set was conducted with 50,000,000 generations and using a random starting tree. Sampled trees were saved every 2,000 generations and the program was allowed to estimate the likelihood parameters required. Results collected prior to stationarity (25–35%) were discarded as burn-in. Convergence was evaluated using the “compare” function in the online application AWTY (Nylander et al., 2008) and using TRACER v1.6 (Rambaut & Drummond, 2007). Fifty-percent Majority Rule consensus trees of all the saved posterior probability trees were computed for each analysis.

### Divergence time estimation

Bayesian divergence time analysis was conducted on the complete cpDNA data set using BEAST v.2.1.2 (Bouckaert et al., 2014). The GTR + I + G model, a lognormal uncorrelated relaxed clock model (Wertheim et al., 2010; Brown & Yang, 2011) and a optimal birth-death model tree prior were imposed in all the analyses. Two fossils were used as prior calibrations for two nodes of the tree following Minaya et al. (2017). Pooid phytholiths described by Zucol et al. (2010) from the Mid-Eocene (Zucol et al., 2010; Strömberg, 2011) and macrofossil remains of a stipoid grass from the Late Eocene (Manchester, 2001; Strömberg, 2011) were used to calibrate the respective crown nodes of Pooideae and Stipeae. These nodes were constrained to have mean ages of 44 ± 4 Myr (mean = 44; *SD* = 1.95) and 36 ± 3 Myr (mean = 36; *SD* = 1.5), respectively (cf. Minaya et al., 2017), and a normal distribution was used in both cases. Although normal distributions are not generally considered suitable for primary calibrations, we consider that the fossils used fulfill the conditions indicated by Ho & Phillips (2009) that make use of a normal prior advisable. Wide ranges and a uniform distribution were imposed for all substitution rates in order to cover most biologically realistic values.

Four independent Markov Chains were run for a total of 600,000,000 generations. The impact of the prior on posterior values was assessed following Drummond & Bouckaert (2015). We used TRACER v1.6 to analyse the log files and to assess convergence through the effective sample size (ESS ≥ 200; Drummond & Rambaut, 2007). Resulting trees from the four searches were combined using LogCombiner v.1.7.2 (Drummond & Rambaut, 2007) with a burn-in of 25%. A maximum credibility tree was constructed using TreeAnnotator v.1.7.2 (Drummond & Rambaut, 2007). Trees were represented using the R package *strap* (Stratigraphic Tree Analysis for Palaeontology; Bell & Lloyd, 2014).

### Diversification rates in the Pooideae

We used MEDUSA (Alfaro et al., 2009) as implemented in the R package Geiger (Harmon et al., 2008; R Development Core Team, 2011) in order to test for changes in net diversification rates in the Pooideae. This approach is based on the equations from the birth-death likelihood model described by Rabosky (2006) and Rabosky et al. (2007). MEDUSA uses the Akaike Information Criterion (AIC) to compare different models, each model including a particular combination of phylogenetic relationships and taxonomic data (species richness of extant groups) with particular values of diversification and relative extinction (Alfaro et al., 2009).

MEDUSA analyses were carried out using a skeleton-phylogenetic tree which was constructed by pruning the original time-calibrated tree with the drop.tip option in the R package APE (Paradis, Claude & Strimmer, 2004; R Development Core Team, 2011). In the analysis, we considered nine out of the twelve Pooideae tribes and supertribes defined by Soreng et al. (2015) (Brachyelytreae, Meliceae, Lygeae, Nardeae, Stipeae, Diarrheneae, Brachypodieae, and the supertribes Poodae and Triticodae, *sensu* Soreng et al., 2015) that were sampled and reconstructed as monophyletic groups in our plastid tree (see Results). One sample per tribe was included in the skeleton tree except for the supertribes Triticodae and Poodae: two samples were added for the Triticodae representing tribes Bromeae and Triticeae and a larger representation of 13 out of 19 subtribes (∼96,6% of the Poodae diversity; cf. Clayton et al., 2006; Soreng et al., 2015) was included for the Poodae. Species diversity in each tribal and subtribal lineage was extracted from Kellogg (2015a) and Soreng et al. (2015), and independent analyses were carried out using diversity matrices obtained from both sources. Diversity values must be considered cautiously, as they may vary as new taxonomic information is generated. Nevertheless, they constitute reasonable proxies for the relative diversity of groups, which is essential in tracing global diversification patterns (e.g., Laenen et al., 2014). In order to incorporate species richness uncertainty in the test, 500 additional analyses were run using diversity matrices built by randomly drawing diversity values for each tribe or subtribe from a data base including: the number estimated as explained above and the maximum and minimum diversity values (the estimate ± 20%). Phylogenetic uncertainty was also accounted for in the analyses by conducting new tests using 500 randomly sampled pruned trees (Drummond et al., 2012). For all analyses, we estimated ΔAICs and net diversification and relative extinction rates. The maximum number of models was set to 47 (sum of the tips and nodes of the trees after pruning to major clades; Escudero & Hipp, 2013) and results were summarized following Escudero & Hipp (2013).

We also used Bayesian Analyses of Macroevolutionary Mixtures (BAMM, Rabosky et al., 2013; Rabosky et al., 2014a; Shi & Rabosky, 2015) to detect and quantify heterogeneity in diversification rates, as implemented in software BAMM v. 2.5 and R package *BAMMtools* (Rabosky et al., 2014b). This approach acknowledges that a complex mixture of dynamic diversification regimes may occur in a single tree. Accordingly, this approach allows speciation and extinction rates to vary both through time and among lineages. To account for incomplete sampling, we specified partial sampling fractions for each subtribe clade (or tribes when subtribes were not used), based on the percentage of species sampled from each subtribe clade. We performed two different analyses. First, we conducted a test in which diversification rates remain constant through time unless a shift in diversification rates is detected (this is basically the same approach implemented in MEDUSA). Second, we performed an analysis in which diversification rates change through time within each diversification regime. In addition, shifts in diversification rates are also allowed. For each analysis we ran BAMM for 10 million generations. We used the R package *coda* (Plummer et al., 2006) to assess MCMC convergence. Finally, *BAMMtools* R package was used to postprocess the BAMM output and to visualize and summarize the parameters of the diversification regimes with highest posterior probabilities.

### Patterns of chromosome number evolution

The chromosomal evolution of the Pooideae was modeled on the Bayesian dated phylogenetic tree produced with BEAST using the software CHROMEVOL v. 2.0 (Mayrose, Barker & Otto, 2010). This program implements a likelihood-based method for tracking the pattern of haploid chromosome number changes along a phylogeny (Mayrose, 2014). This analysis aims at reconstructing ancestral haploid chromosome numbers, but it makes no reference (and it does not require) the calculation of the inferred chromosome base number *x* (Mayrose, Barker & Otto, 2010; Cusimano, Sousa & Renner, 2012). In our study, haploid chromosome numbers for the sampled species were coded following the “informed” coding scheme proposed by Cusimano, Sousa & Renner (2012). We took into account phylogenetic information on the different genera included in the analysis, and we assigned chromosome numbers found in the early diverging species to the entire genus (Cusimano, Sousa & Renner, 2012). When no precise phylogenetic information was available for a genus, the lowest haploid chromosome number was applied. This coding scheme allowed us to deal with the problem of the existence of different ploidy levels in a species and also the low-density sampling conducted in most Pooideae taxa. Chromosome data were taken from our own records and from public databases and literature (e.g., Goldblatt & Johnson, 1979; Watson & Dallwitz, 1992; Catalán et al., 2004; Gillespie & Soreng, 2005; Winterfeld, Döring & Röser, 2009; Winterfeld, Perner & Röser, 2011; Bennett & Leitch, 2012; *Tropicos.org*; Table S2). The input tree was pruned to eliminate all terminals for which chromosome numbers were unknown using the package APE (126 terminals were included in the analysis). The ten models of chromosome evolution in CHROMEVOL v. 2.0 are based on different combinations of three to six of the following eight parameters of rates of chromosome mutation: (1) whole genome duplication; (2) demi-polyploidization (half-duplication of the chromosome number through auto- or allopolyploidization; Mayrose, Barker & Otto, 2010); (3) gain or (4) loss of a single chromosome (dysploidy); (5) gain or (6) loss of a single chromosome when ascending or decreasing dysploidy rates depend on the current number of chromosomes; (7) an optimized chromosome number which characterizes a phylogenetic group; and (8) rate for transitions by base number (Mayrose, Barker & Otto, 2010; Glick & Mayrose, 2014). The fit of each of the ten available models was investigated using the AIC (Bournham & Anderson, 2002). Our analysis focused on the better sampled core pooids (including the supertribes Triticodae and Poodae). All taxa from this group for which chromosome numbers from early diverging lineages were found were included.

## Results

### Phylogenetic reconstruction and divergence times

The number of sequences used in the analysis, the number of aligned bases per gene and the percentage of parsimony informative characters in each data set are summarized in Table S1. A higher average number of parsimony informative positions was observed in coding than in non-coding regions (51.01% *vs* 31.21%), although the difference was not significant. The topologies recovered in the Bayesian (MrBayes and BEAST) and parsimony trees (Figs. S1 and S2) were congruent and only the Bayesian topology recovered with BEAST will be discussed.

Bayesian phylogenetic trees from the different cpDNA data sets were congruent (results not shown) in topology, although trees based on coding regions (*mat*k, *ndh*F) recovered higher posterior probability values. Special attention was paid to the *trnH*-*psbA* plastid region since micro-inversions have been detected by different authors (e.g., Romaschenko et al., 2012). No supported differences in topology were found in the *trnH*-*psbA*-based tree with respect to the other plastid topologies and overall support values in the global plastid tree increased when the *trnH*-*psbA* region was considered.

**Figure 1:**
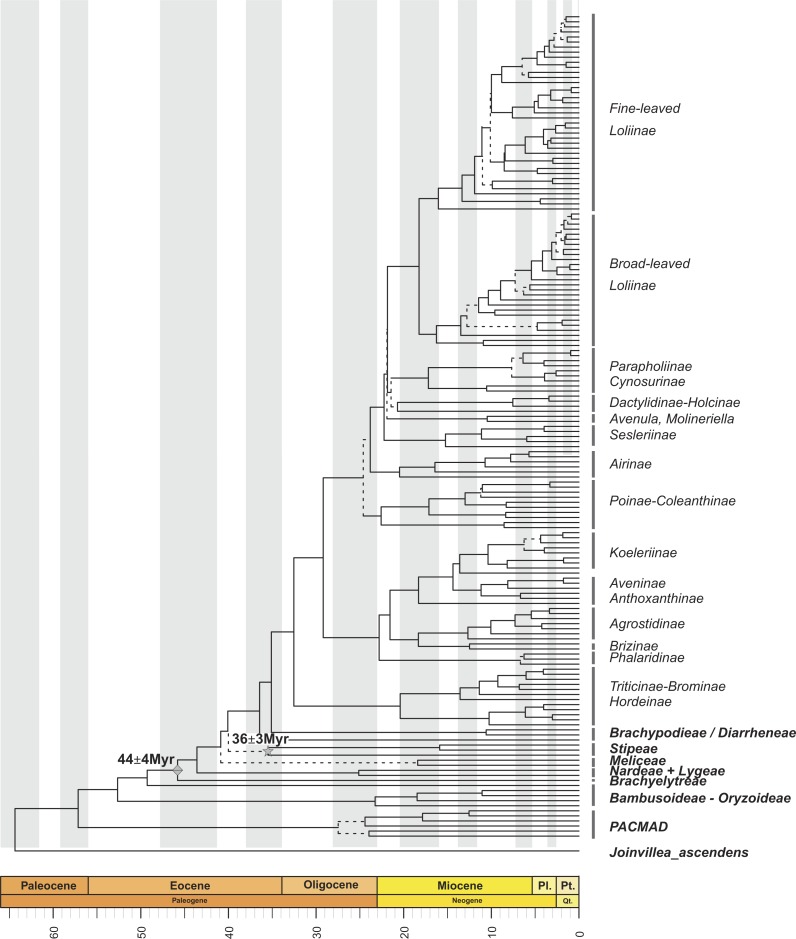
Maximum clade credibility tree from the Bayesian analysis of plastid DNA sequences (*trnH-psbA*, *trnT-L*, *trnL-F*, *ndhF* and *matk*). Maximum clade credibility tree from the Bayesian analysis of plastid DNA sequences (*trnH-psbA*, *trnT-L*, *trnL-F*, *ndhF* and *matk*) from 163 samples representing 152 Pooideae, 6 PACMAD, 3 Bambusoideae and 1Oryzoideae grass species and *Joinvillea ascendens* as outgroup, constructed with BEAST using a Yule prior. Divergence times were inferred using a relaxed molecular clock. Dashed lines represent branches with *PPS* < 0.8. Mes., Mesozoic; Cr., Cretaceous; Oligo., Oligocene; Pl., Pliocene; Pt., Pleistocene; Qt., Quaternary. Diamond and star symbols indicate the fossil-based calibration priors imposed to the crown nodes of Pooideae and Stipeae, respectively.

The concatenated data set built using the five plastid regions included 163 taxa (Appendix S1, Table S1) and 5,588 characters, 26% of which (1,505) were parsimony informative. Within the BOP clade (Bambusoideae, Oryzoideae, Pooideae; Clark, Zhang & Wendel, 1995), the Oryzoideae was sister to a clade including the Pooideae and the Bambusoideae with moderate support (Posterior probability support -PPS- 0.92; Fig. 1). In the PACMAD clade there was a split of two highly supported lineages (PPS 1.0), one including Danthonioideae and Chloridoideae and another comprising Panicoideae (including Centothecoideae, Sanchez-Ken & Clarke, 2010) and Arundinoideae.

Within the Pooideae, all tribes defined by the GPWG (2001) but Stipeae were recovered as monophyletic with strong support (Fig. 1; Fig. S1). According to our estimations, the split of the pooids took place from the Mid-Eocene onwards. The ancestor of the early diverging lineage leading to the Brachyelytreae (PPS 1.0) (i.e., Most Recent Common Ancestor (MRCA) of Pooideae), likely originated around 45.5 Myr (High Posterior Density (HPD) 42.5–48.1 Myr). The estimated ages of the MRCAs of the consecutive splits leading to Nardeae *s.l.* (*sensu* Schneider et al., 2009, including Lygeae) (43.2 Myr , (HPD, 39.7–45.6 Myr), PPS 0.98), Meliceae (40.3 Myr (HPD 38.2–44.3 Myr), PPS 0.99) and Stipeae (with low support, 39.4 Myr (HPD 34.9–43.2 Myr), PPS 0.3) were dated in the Mid to Late Eocene.

Most of the Pooideae lineages branched off during the Oligocene and Miocene (Fig. 1; Fig. S1). The MRCAs of Diarrheneae (37 Myr (HPD 32.8–41.6 Myr), PPS 0.98) and Brachypodieae (35.7 Myr (HPD 31.1–40 Myr), PPS 0.95) were estimated to have diverged in the Late Eocene-Early Oligocene (Fig. 1), those of Triticodae and Poodae (33.5 Myr (HPD 29.2–38.3 Myr), PPS 1) in the Early Oligocene, and those of Bromeae and Triticeae in the Early to Mid-Miocene (21.3 Myr (HPD 14.6–28.7 Myr), PPS 0.99).

Within the Poodae, the split of the sister “Aveneae-type cpDNA” and “Poeae-type cpDNA” lineages (*sensu* Soreng et al., 2003; Soreng et al., 2007; Soreng et al., 2015) was inferred to have occurred in the Early Oligocene (30.6 Myr (HPD 25.9-34.9 Myr), PPS 0.99). Within the former clade, our data supported the early divergence of the Phalaridinae lineage in the Late Oligocene (24.1 Myr (HPD 18.9–29.5 Myr), PPS 0.97), then the split of Agrostidinae and Brizinae (including *Airopsis* Desv.), and then the more recent divergence of Koeleriinae and Aveninae in the Mid-Miocene (15.4 Myr (HPD 11.2–19.8 Myr), PPS 0.99). Within the second clade our analysis inferred the successive splits of the Poinae / Coleanthinae (26.2 Myr (HPD 19.6–27.5 Myr), PPS 0.97) and the Airinae (25.3 Myr (HPD 21.4–29.3 Myr), PPS 0.80) in the Late Oligocene, and those of the Sesleriinae (including *Mibora* Adans.) (23.7 Myr (HPD 20–27.7 Myr), PPS 0.95) and the Loliinae (22.9 Myr (HPD 19.3–26.5 Myr), PPS 0.96) in the Late Oligocene-Early Miocene (Fig. 1; Fig. S1). Our results supported an Early Miocene (20 Myr (HPD 15.9–23.7 Myr)) divergence for the well supported broad-leaved and fine-leaved lineages of Loliinae (Catalán et al., 2004). Diversification within these clades took place mostly between the Late Miocene and the Pleistocene; and their phylogenetic reconstruction fully agreed with Inda et al. (2008), Inda et al. (2013) and Minaya et al. (2017).

**Figure 2:**
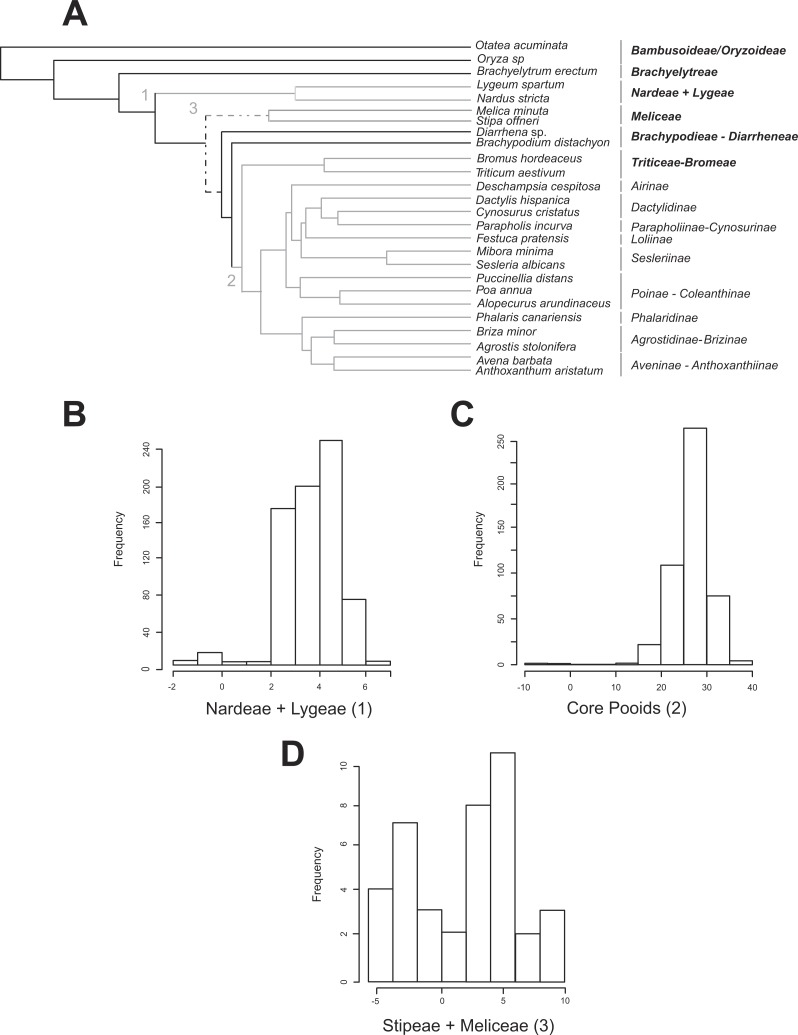
Shifts in diversification detected for the Pooideae using the MEDUSA approach. (A) Pruned (26 terminals) ultrametric Bayesian consensus tree obtained with BEAST (for details see ‘Materials and Methods’). Gray branches represent significant shifts from the background rate as estimated with the algorithm MEDUSA using 500 diversity data sets obtained from Soreng et al. (2015; for details see ‘Materials and Methods’). Shift numbers are indicated on branches: 1, Nardeae and Lygeae; 2, Core pooids; 3, Meliceae + Stipeae. Numbers as in Fig. 4. Dashed lines represent branches with support below 0.8 PPS. (B–D) Histograms showing the diversification rate analysis conducted with MEDUSA using 500 random Bayesian trees and the diversity data set based on Soreng et al. (2015; for details see ‘Materials and Methods’). Each histogram represents a significant shift in diversification rates. B, Nardeae + Lygeae; C, Core pooids; D, Stipeae + Meliceae. The *X*-axis represents the amount of change in the AIC value. The *Y*-axis represents the frequency of the rate shift (number of trees where the change is registered). Names of lineages correspond to the most updated tribal classification of the Pooideae (Soreng et al., 2015). Shift numbers are indicated in the histograms.

### Rates of diversification in the Pooideae. Evolutionary shifts

**Figure 3:**
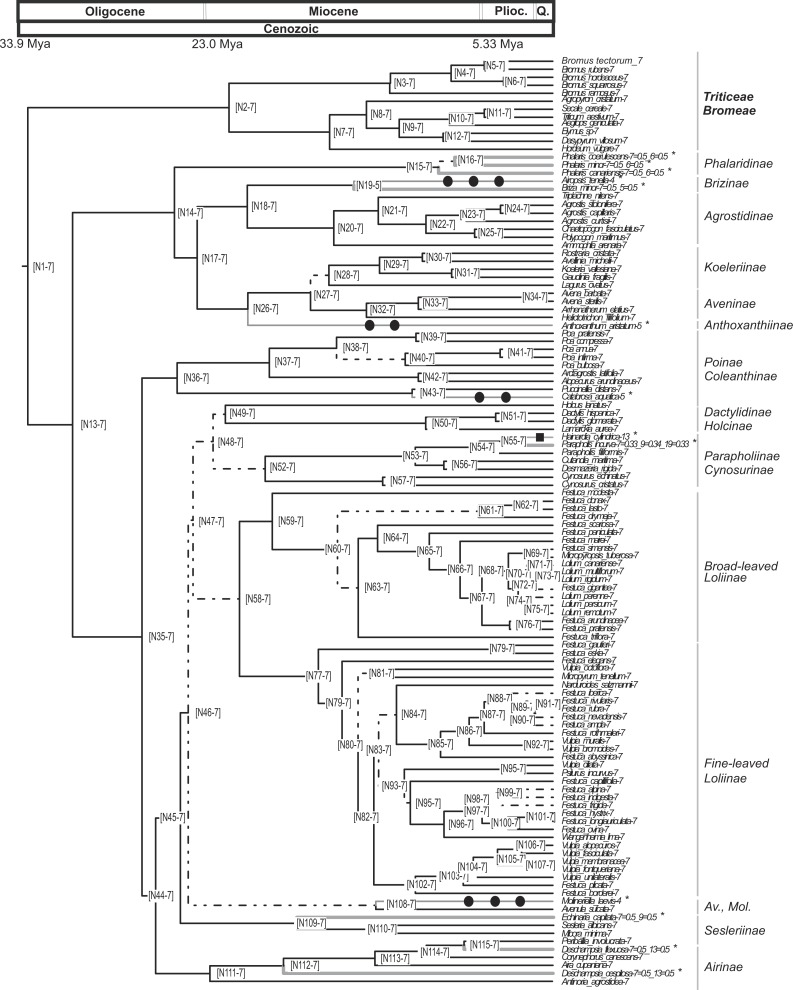
Pruned ultrametric MCC tree produced with BEAST. Pruned ultrametric MCC tree produced with BEAST (for details see ‘Materials and Methods’). Gray thin branches represent significant changes in haploid chromosome number from the background value *n* = 7 detected using CHROMEVOL. Affected terminals are indicated with an asterisk. Filled square, transition in base number; filled circle, haploid chromosome number transition based on single chromosome loss. Dashed lines represent branches with *PPS* < 0.9. Thick gray lines represent events not accounted for in the simulation. Plioc, Pliocene. Q, Quaternary.

All MEDUSA analyses conducted were largely congruent, and only results using Soreng et al. (2015) diversity data and integrating diversity and phylogenetic uncertainty will be discussed (see ‘Materials and Methods’). Our results based on 500 random diversity data sets and one pruned phylogenetic tree recovered three significant (ΔAIC greater than 2.0, gray branches, Fig. 2A) shifts from the background diversification rate (net diversification rate = *r* = 0.098(*SD* = 0.0022) spp. Myr^−1^, relative extinction rate = ε = 0.271(*SD* = 0.344)): (1) a weakly supported decrease in net diversification rates affecting the Nardeae+ Lygeae (*Nardus* L. + *Lygeum* Loefl.): 248 out of 500 analyses showed decreased net diversification rates (ΔAIC = 2.063; *r* = 0.0159(*SD* = 0.001) spp. Myr^−1^, ε = 3.700 •10^−7^ (*SD* = 0.00)); (2) a strongly supported increase in net diversification rates affecting the lineage comprising the core pooids (Triticodae + Poodae): 490 out of 500 analyses showed increased net diversification rates (ΔAIC = 22.41; net diversification rate = *r* = 0.277(*SD* = 0.005) spp. Myr^−1^, relative extinction rate = ε = 0.021(*SD* = 0.0755)); (3) a moderately to strongly supported increase in net diversification rates in the Meliceae + Stipeae: 381 out of 500 tests showed increased net diversification rates (ΔAIC = 4.42; *r* = 0.1896 (*SD* = 0.004) spp. Myr^−1^, ε = 0.00014 (*SD* = 1.66 •10^−4^)). The tests conducted using one diversity matrix and 500 pruned phylogenetic trees (Figs. 2B–2D) also recovered significant deviations from the background diversification rates (*r* = 0.127 (*SD* = 0.0008) spp. Myr^−1^, ε = 0.0041 (*SD* = 0.035): (1) Nardeae + Lygeae (435 out of 500 trees showed decreased net diversification rates; ΔAIC = 3.92; *r* = 0.0139 (*SD* = 0.033) spp. Myr^−1^, ε = 0.063 (*SD* = 0.02); Fig. 2B); (2) the core pooids (460 out of 500 trees showed highly increased net diversification rates; ΔAIC = 26.74; *r* = 0.227 (*SD* = 0.029) spp. Myr^−1^, ε = 0.025 (*SD* = 0.033); Fig. 2C) and (3) Stipeae + Meliceae (30 out of 500 trees showed increased net diversification rates; ΔAIC = 2.0; *r* = 0.147 (*SD* = 0.015) spp. Myr^−1^, ε = 0.00032 (*SD* = 0.000012); Fig. 2D).

BAMM analyses supplied results that were mostly congruent with the results obtained from MEDUSA analyses. We report the results of the diversification model with the highest posterior probability. Two evolutionary regimes with one shift in diversification rates were preferred when diversification rates were constrained to remain constant within the regimes. This model inferred a background regime with initial speciation and extinction rates of 0.17733 spp. Myr^−1^ and 0.07696 spp. Myr^−1^ (net diversification = 0.10037 spp. Myr^−1^), respectively, and a shift in diversification at the origin of the core pooids (supertribes Poodae + Triticodae) clade with initial speciation and extinction rates of 0.61166 spp. Myr^−1^ and 0.39408 spp. Myr^−1^ (net diversification = 0.21758 spp. Myr^−1^), respectively. Three evolutionary regimes with two shifts in diversification rates were preferred when speciation rates were allowed to change within each regime. This model inferred a background regime with initial speciation and extinction rates of 0.51053 spp. Myr^−1^ and 0.55409 spp. Myr^−1^, respectively, and a growth parameter of speciation rates of 0.01308. A first shift in diversification at the origin of the clade of the sister lineages Nardeae and Lygeeae (Nardeae *s.l. sensu* Schneider et al., 2009) with initial speciation and extinction rates of 0.08383 spp. Myr^−1^ and 0.10388 spp. Myr^−1^, respectively, and a decay parameter of speciation rates of −0.00237. And a second shift in in diversification at the origin of the clade of the tribe Brachyelytreae with initial speciation and extinction rates of 0.09514 spp. Myr^−1^ and 0.20010 spp. Myr^−1^, respectively, and a decay parameter of speciation rates of −0.00099.

### Evolution of chromosome numbers in the core pooids

The analysis conducted using CHROMEVOL (Mayrose, Barker & Otto, 2010) on the core pooids showed that the best-fitting model of chromosome number evolution indicated an underlying haploid chromosome number of seven (not necessarily the chromosome number at the root of the core pooids; Mayrose, 2014). The selected model (AIC: 92.294) included the following parameters: base number (a specified chromosome number that characterises a phylogenetic group; Mayrose, 2014), transitions by base number given the base number of the phylogeny (Mayrose, 2014) and gain and loss of single chromosomes (For the AIC values of the other models supported by the program see Table S3). Our results indicated that in the core pooids the loss of single chromosomes is by far the most frequent chromosome mutation (although with a low prevalence; single chromosome loss = 0.00618 mutations/Myr), whereas the transitions by base number show a rate of transition (0.000289 mutations/Myr) an order lower than the loss of single chromosomes. The analysis recovered evidence for 11 chromosome number transitions that occurred over the last 20 million years: one transition in the haploid chromosome number (1 transition by base number) and 10 chromosome losses (Fig. 3). Losses of single chromosomes were inferred to be independent of the current chromosome numbers in the lineages. Putative chromosome mutation events affecting several taxa (*Phalaris coerulescens* Desf., *Phalaris minor* Retz. and *Phalaris canariensis* L.; *Briza minor* L., *Parapholis incurva* (L.) C.E.Hubb., *Echinaria capitata* (L.) Desf., *Deschampsia flexuosa* Trin. and *Deschampsia cespitosa* (L.) P.Beauv.) were not accounted for in the simulations (expectations below 0.5). Nevertheless, all of them were single drops in base chromosome number.

**Figure 4:**
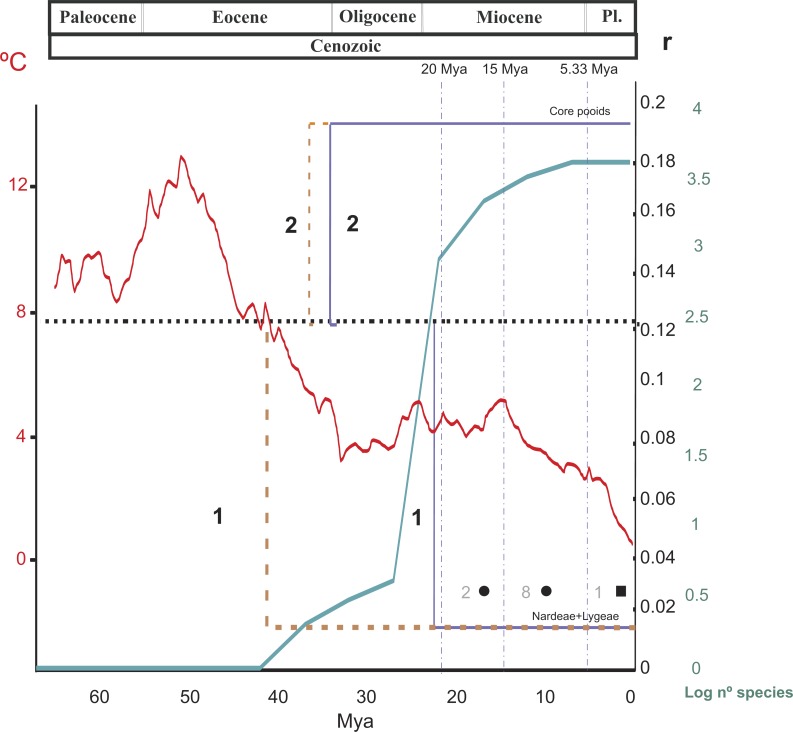
Summary of the results of the diversification rate analysis and the chromosome evolution analysis. Summary of the results of the diversification rate analysis and the chromosome evolution analysis across different temporal slices (TS) related to the divergences of the main Pooideae lineages. TSI, before 20 Myr; TSII, 20–15 Myr, TSIII, 15–5.33 Myr, TSIV, 5.33 Myr-present. 1 and 2 indicate shifts in diversification rates affecting, respectively, the Nardeae *s*.*l*. (Nardeae + Lygeae) and the core pooids (Triticodae + Poodae). The black dashed line represents the background diversification rate estimated from 500 ultrametric trees randomly selected from the BEAST results (*r* = 0.127; see ‘Materials and Methods’). For each case (1–2), the brown and blue lines represent shifts in diversification rates of, respectively, their stem and crown nodes. Shifts affecting the poorly supported Meliceae + Stipeae are not represented in the graphic. The green line represents the number of species (log scale) estimated in the crown nodes of the studied Pooideae tribal and subtribal lineages. The red line represents temperatures during the Cenozoic. Chromosome evolution (in dark gray): Filled square, haploid chromosome base number transition based on duplication (polyploidy); filled circle, single chromosome losses. Numbers refer to the number of events at each temporal window. Pl, Pliocene.

## Discussion

### Phylogeny and tempo of divergence of the Pooideae and the core pooids

The recovered topologies (Fig. 1; Fig. S1) largely supported the lineage arrangements proposed for the Poaceae by previous authors (e.g., GPWG, 2012; Soreng et al., 2015; Kellogg, 2015a; Sancho et al., in press). The exception to this is the moderate to poorly supported sister relationship recovered between the Pooideae and the Oryzoideae in most analyses (Fig. 1, Figs. S1, S2).

The dates inferred in our analysis for the onset of the diversification of the Pooideae, as well as for the divergences of its main lineages were roughly consistent with previous results by other authors (e.g., Vicentini et al., 2008; Bouchenak-Khelladi et al., 2009; Sancho et al., in press). The successive splits of the early-diverging pooid lineages (Brachyelytreae, Nardeae *sensu lato* (Schneider et al., 2009), Meliceae, Stipeae, Diarrheneae and Brachypodieae; Fig. 1) concurs with the Late Eocene-Oligocene climate transition to a cooler and drier climate that favored the expansion of grasslands (Zhonghui et al., 2009; Edwards et al., 2010; Strömberg & McInerney, 2011). Remarkably, our phylogenetic results support the controversial intermediate positions of Diarrheneae, as sister to the Brachypodieae + core pooids lineage, and of Brachypodieae as sister to core pooids clade (Davis & Soreng, 2007; Schneider et al., 2009; Soreng et al., 2015; Sancho et al., in press).

Net diversification rates (from MEDUSA) and chromosome transitions for the Pooideae during the Cenozoic, as recovered in this work, are summarized in Fig. 4. Average deep-sea temperatures (Beerling & Royer, 2011) and the increase in species diversity in the subfamily in the last 65 million years have also been included in this figure. Diversification rates were estimated using different methods and combinations of phylogenetic and diversity data (see ‘Materials and Methods’), offering congruent results. This congruence highlights the robustness of the methodology used in the face of uncertainty in diversity data (Feldberg et al., 2014; Laenen et al., 2014).

Background net diversification rates for the Pooideae (*r*) ranged between 0.098 and 0.127 spp. Myr^−1^ (tests based on the pruned consensus tree + 500 diversity matrices *vs* tests applied on 500 post burn-in pruned trees) in MEDUSA analysis and 0.10037 spp. Myr^−1^ in BAMM analysis constrained to constant diversification rates within regimes, which is consistent with the rate estimated for the Poales by Magallón & Sanderson (2001; *r* = 0.1013). In MEDUSA analysis, we found three different putative deviations from the background net rate of diversification in the Pooideae, regardless of the analysis considered. One of these deviations (a decrease in the rate) affected the early-diverging, highly isolated pooid lineage of Nardeae *s. l*. (i.e., Nardeae + Lygeae) (*r* = 0.016 and *r* = 0.014; *SD* = 0.002 and 0.003 in the consensus tree + 500 diversity matrices and the 500 post burn-in trees analyses, respectively; Figs. 2A, 2B–2D). In BAMM analysis—with speciation rates allowed to change within regimes-, we found also a shift in diversification rates in this clade. This shift dated back to the Mid Eocene-Early Oligocene (stem to crown node, Fig. 4). This date predates that of the bursting diversification of grasses that took place in the Oligocene-Miocene resulting in the adaptation of the Pooideae to open habitats (Bouchenak-Khelladi et al., 2010), and could explain the current extraordinarily small diversity of these two monotypic tribes, adapted to opposite ecological conditions (moist habitats Nardeae, aridic saline-soil habitats Lygeae). Another shift (an increase in the rate) was detected for the Meliceae + Stipeae (*r* = 0.016 and *r* = 0.014; *SD* = 0.002 and 0.003 in the consensus tree + 500 diversity matrices and the 500 post burn-in trees analyses, respectively; Figs. 2A, 2B–2D), agreeing with the present taxonomic richness of these pooid tribes (Kellogg, 2015a). However, support for the Meliceae-Stipeae stem branch is low in our phylogenetic analyses (Fig. 1, 2A) and no significant conclusions can be drawn from this result. In BAMM analysis—with speciation rates allowed to change within regimes-, we also found also a shift in diversification rates in the clade of tribe Brachyelytreae but this was not detected in MEDUSA analyses.

Within the core pooids our results are consistent with those of Schneider et al. (2009), detecting three lineages within Triticodae, that correspond to subtribes Triticinae, Hordeinae and Brominae (Fig. S1). Our dating suggests an earlier radiation of the Triticinae (14.2 Myr (HPD 8.5–20.4 Myr)) as compared to that of the Brominae (11 Myr (HPD 6.1–17.3 Myr)) in the Miocene (Fig. 1). Our tree also reconstructs a large Poodae lineage that includes former Aveneae, Poeae, Hainardieae, Phalaridae, Phleeae and Seslerieae (*sensu* Tzvelev, 1976) representatives. Within Poodae the split between the Aveneae-type and the Poeae-type cpDNA lineages (Fig. 1; Quintanar, Castroviejo & Catalán, 2007; Saarela et al., 2010) was estimated to have occurred in the Early Oligocene (30.6 Myr (HPD 25.9–34.9 Myr); Fig. 1), whereas lineages within these large clades radiated from the Mid-Miocene to the Pleistocene (Fig. 1). The phylogenetic relationships recovered for lineages within these groups are consistent with previous data (e.g., Soreng et al., 2003; Quintanar, Castroviejo & Catalán, 2007; Gillespie et al., 2008). Our divergence time estimations suggest an early Miocene origin for *Festuca* L. and its closest allies (20 Myr) that predates the estimation of Inda et al. (2008; 13 Myr) but is similar to that of Minaya et in press al. (2017; 22.5 Myr).

Diversification within the core pooids was especially active from the Late Oligocene to the Pleistocene, which is congruent with the expansion process of C3 temperate Eurasian grasses that began in the Early Oligocene (e.g., Bouchenak-Khelladi et al., 2010; Edwards et al., 2010). A clearly significant shift in net diversification rates was detected for this group (*r* = 0.241, consensus tree analysis + 500 diversity matrices, and *r* = 0.1921, analysis based on 500 post burn-in trees; *SD* = 0.005 and 0.029; Figs. 2A, 2B–2D). In BAMM analysis -constrained to constant diversification rates within regimes-, we found also a clear shift in diversification rates in this clade (up to 0.21758 spp. Myr^−1^). Our results indicate a temporal coincidence between the increase in the rate of diversification detected in the core pooids and the drop in global temperatures that took place in the Middle to Late Eocene and the Oligocene (Beerling & Royer, 2011). Interestingly, this increase in diversification of the mostly temperate core pooids occurred before the divergence and diversification of the ungulate families Bovideae and Cervideae in moist Eurasian regions, which took place in the Late Oligocene (Matthee & Davis, 2001; Bouchenak-Khelladi et al., 2009). By contrast, diversification of tropical, mostly C4, PACMAD grasses concurred mostly with the diversification of some mamalian herviborous lineages like Antilopinae *s.l.*, Hippotragineae and Alcelaphineae within the Bovidae (Bouchenak-Khelladi et al., 2009).

Several authors have highlighted the connection between the development of a cooler, dryer climate in the Oligocene and the diversification of the pooid grasses in recent years (Kellogg, 2001; Bredenkamp, Spada & Kazmierczak, 2002; Strömberg, 2005; Edwards et al., 2010; Strömberg & McInerney, 2011). The same pattern has been discovered for other highly diverse herbaceous groups such as the Cyperaceae (Escudero et al., 2012; Escudero & Hipp, 2013). The diversification of the entirely C3 core pooids during the Oligocene continued during the Miocene and the Pliocene (Fig. 1) and developed into primary grasslands in both hemispheres (Bouchenak-Khelladi et al., 2009; Edwards et al., 2010).

The number of diversification shifts detected for the C4 grass lineages in a genus-level phylogenetic analysis (*n* = 800) of the PACMAD group was much higher and occurred in more recent times (24 shifts during the Pliocene and the Miocene according to Bouchenak-Khelladi et al., 2009) as compared to the Pooideae. This could be explained, at least partially, by differences in the methodology, sampling and evolutionary scale of the analyses (see Bouchenak-Khelladi et al., 2009). However, no shift older than 23 (18.2–27.8) Myr was detected in the PACMAD despite the much older origin of the group (Late Eocene; Bouchenak-Khelladi et al., 2009). Additionally, no shifts younger than the boundary between the Eocene and the Oligocene were detected in our analyses despite the fact that 27 (14 tips and 13 nodes) of the 46 (23 tips and 25 nodes) analyzed clades are younger than this boundary. This difference could be explained by the heterogeneous expansion and diversification of the C4 grasses, triggered mostly by local ecological factors and disturbances rather than by changes in atmospheric conditions (Osborne & Beerling, 2006). According to Tipple & Pagani (2007) and Edwards & Still (2008), this ecological heterogeneity in the Miocene mostly affected warm parts of the world, where pooid grasses were less represented. Our results show that the temperate core pooids have presented a high and relatively constant diversification rate correlated with (and possibly influenced by) the atmospheric conditions in temperate areas (Fig. 4). We have also observed a gap between the taxonomic diversification in Pooideae that started in the Mid Eocene-Early Oligocene (Fig. 1) and their rise to ecological dominance today, mostly in the Northern Hemisphere. Strömberg (2005) found a similar pattern in the Cenozoic of North America. This observation supports the idea that the diversification of grasses was prior to the opening of new ecological opportunities derived from the Neogene climatic deterioration (Strömberg, 2005).

### Chromosome evolution in the core pooids

Chromosome transitions are considered key mechanisms in angiosperm evolution (e.g., Soltis et al., 2009). Different events are included in these mechanisms, mainly polyploidy (including polyploidization and demi-polyploidization *sensu* Mayrose, Barker & Otto, 2010), gains, and losses of single chromosomes (Cohlan et al., 2005). Transitions, especially polyploidy (in the broad sense) are widespread in angiosperm evolution (e.g., Soltis et al., 2009; Mayrose et al., 2011), and their impact in diversification has been widely disputed (Soltis et al., 2009; Arrigo & Barker, 2012, and references therein, but see also Soltis et al., 2014). Recent reviews of the methodologies used to assess the connection between polyploidisation and diversification indicate that such relationship is ambiguous (Kellogg, 2016).

Our analyses show that the underlying haploid chromosome number (*n* = 7) is remarkably constant throughout the core pooid phylogenetic tree (Fig. 3). This number is consistent with the literature (e.g., Tzvelev, 1989; Shchapova, 2012) and represents a derived state in the family (Salse et al., 2008). Our analysis detected 11 chromosome changes throughout the phylogeny (Figs. 3 and 4). More specifically, we detected 10 single chromosome losses (87.5%) and one polyploidization (transition by base number; all intrageneric polyploidisation events were excluded from our analysis; Cusimano, Sousa & Renner, 2012). All changes were restricted to middle to shallow levels of the tree, up to the Pliocene-Pleistocene (21 Myr-present; Figs. 3 and 4).

The prevalence of polyploidy in the core pooids has been highlighted by several authors (e.g., Hsiao et al., 1995; Catalán, Kellogg & Olmstead, 1997), with allopolyploidy being especially important in the grasses (e.g., Stebbins, 1971; Levy & Feldmann, 2002; Roodt & Spies, 2003; Kellogg, 2015a). Our analyses failed to register polyploidization events that led to the origin of new genera (with the possible exceptions of *Arctagrostis* Griseb. and *Ammophila* Host.) and we did not find a direct relationship between the shifts in diversification and polyploidization (Fig. 4). However, the question remains open since shallow parts of the phylogeny were not included in our diversification analyses that were performed without full sampling of extant species. Nevertheless our findings would reinforce the idea that newly formed polyploid lineages in the core pooids might experience higher extinction risk and fail to persist, as described in other angiosperm lineages (e.g., Fawcett, Maere & Van de Peer, 2009; Mayrose et al., 2011; Escudero et al., 2014; but see Soltis et al., 2014, and Kellogg, 2016), or that the analysis is not well suited for hybrid allopolyploid scenarios (Soltis et al., 2014; Kellogg, 2016). It is important to consider, however, that we are assuming (for genera with unclear phylogenies; e.g., *Koeleria* Pers., *Parapholis* C.E.Hubb.), that the lowest chromosome number in the group represents the earliest branching lineage. Besides, very diverse genera that are entirely polyploid (e.g., *Calamagrostis*, *Elymus*; Hilu, 2006; Kellogg, 2015a) have not been included in our analysis. The existence of allopolyploid clades seems to indicate that in some instances there is an association between polyploidy and rapid diversification, supporting polyploidy as an evolutionary driving force in some specific (generic) lineages of the core pooids, as suggested by Stebbins (1985). To what extent that trend applies to taxonomic levels above the species is disputed (Mayrose et al., 2011; Soltis et al., 2014).

Our results must be interpreted cautiously due to limitations in the analysis as well as in our data set. Chromevol tracks changes along a phylogeny where relationships are expressed as dichotomies in a phylogenetic tree. It has not been designed to analyze reticulate evolution scenarios involving allopolyploidy (Soltis et al., 2014; Kellogg, 2016), common in the Pooideae (e.g., Winterfeld et al., 2014; Kellogg, 2015a). This is one of the main criticisms of the method and might affect the precision of its estimates, especially since the effect of polyploidy on topologies is not well understood (Soltis et al., 2014). As noted by Mayrose et al. (2014), this criticism applies to most comparative methods using phylogenies. By using uniparentally inherited plastid markers, we expect a fully bifurcating phylogeny even in the face of widespread interspecific hybridization (Mayrose et al., 2014). However, maternally-inherited plastid markers are also prone to topological conflicts in those cases where bidirectional crosses have resulted in the same allopolyploid species, like reported in several pooids (Catalán et al., 2016, and references therein).

## Conclusions

The phylogenetic tree obtained was largely congruent with previously published results. Diversification of the BOP clade took place between the Middle to Late Paleocene onwards and tribes Diarrheneae and Brachypodieae were shown as intermediate between the early diverging basal pooids (Brachyelytreae, Nardeae, Meliceae+Stipeae) and the more recently evolved core pooids (Poodae, Triticodae). Early divergence seems to be correlated with the expansion of grasslands due to climate changes in the Late Eocene-Oligocene.

Net diversification rates within the Pooideae were not constant, and one to three strongly to weakly supported shifts were detected, affecting the core pooids (Poodae + Triticodae) and the tribes Nardeae, Stipeae + Meliceae and Brachyelytreae. The shift in the core pooids was dated back to the Late Oligocene-Early Miocene, which is consistent with the drop in global temperatures and the expansion of C3 temperate Eurasian grasslands.

No links were found between chromosome transitions and major diversification events in the core pooids, as chromosome mutations were mostly restricted to shallow parts of the phylogeny. The base haploid chromosome number (*n* = 7) was remarkably stable in the core pooids phylogeny, representing a derived state in the family.

##  Supplemental Information

10.7717/peerj.3815/supp-1 Appendix S1 List of taxa included in this study Systematic and phylogenetic adscriptions follow those proposed by Soreng et al., 2003, Soreng et al., 2007, Soreng et al., 2015, and Torrecilla & Catalán, 2002, Torrecilla, López Rodríguez & Catalán, 2004, Catalán, 2006, Quintanar, Castroviejo & Catalán, 2007, Bouchenak-Khelladi et al., 2008, Inda et al., 2008, Schneider et al., 2009, and Minaya et al., 2017. Herbarium codes of official herbaria follow *Index Herbariorum* (Thiers, consulted June the 16th 2017). UZ, University of Zaragoza (Spain) Herbarium. An asterisk was added to the sequences that were downloaded from Genbank.

10.7717/peerj.3815/supp-2 Appendix S2 Expanded materials and methods Procedures for DNA isolation, DNA amplification and sequencing, and for sequence alignment.

10.7717/peerj.3815/supp-3 Table S1 Number of samples sequenced per region Number of samples sequenced per region (one sample per species); size of the alignment; percentage of parsimony informative characters (p.i.); consistency index (CI) and retention index (RI) (excluding non informative characters).

10.7717/peerj.3815/supp-4 Table S2 Haploid chromosome number for species included in the study Haploid chromosome number for species included in the study based on: Catalan et al., 1997, Catalán et al., 2004, Díaz-Pérez *et al*., 2014, Döring et al., 2007, Escobar *et al*., 2011, Essi et al., 2008, Fortune et al., 2008, Gillespie et al., 2005, Gillespie et al., 2008, Goldblatt & Johnson, 1979, Inda et al., 2013, Kellogg et al., 2015, Minaya et al., 2015, Peterson et al., 2006, Pimentel et al., 2013, Quintanar, Castroviejo & Catalán, 2007, Quintanar *et al*., 2010, Saarela et al., 2010, Saarela et al., 2015, Schneider et al., 2011, Schneider et al., 2012, Soreng et al., 2007, Soreng et al., 2010, Soreng et al., 2015, Voshell *et al*., 2011, Watson & Dallwitz, 1992, Winterfeld, Perner & Röser, 2011, Winterfeld et al., 2014. Hp., haploid.

10.7717/peerj.3815/supp-5 Table S3 Models tested, parameters included and AIC values obtained *per* model in the chromosome base number evolution analysis conducted using CHROMEVOL 2.0 Models tested, parameters included and AIC values obtained *per* model in the chromosome base number evolution analysis conducted using CHROMEVOL 2.0 [for a full description of the models and parameters see Mayrose (2014)]. G(ConstR), rate for ascending dysploidy (single chromosome gain); L(ConstR), rate for descending dysploidy (single chromosome loss); BN(R), rate for transitions by base number; BN, base number, a specified chromosome number that characterizes a phylogenetic group; D(ConstR), rate for whole genome duplication; DP(R), rate for demi-duplication (a multiplication of the chromosome number by a factor of 1.5); G(LinearR), rate for ascending dysploidy dependent on the current chromosome number; L(LinearR), rate for descending dysploidy dependent on the current chromosome number.

10.7717/peerj.3815/supp-6 Figure S1 Bayesian 50% majority rule consensus tree obtained in the analysis of plastid DNA sequences Bayesian 50% majority rule consensus tree obtained in the analysis of plastid DNA sequences (*trnH-psbA*, *trnT-L*, *trnL-F*, *ndhF* and *matk*) from 163 samples representing 152 Pooideae, 6 PACMAD, 3 Bambusoideae and 1 Ehrhartoideae grass species and * Joinvillea ascendens* as outgroup, constructed with MrBayes 3.2.2. Dashed lines represent branches with posterior probability (*PP*) < 0.9. // indicates shortened branches.

10.7717/peerj.3815/supp-7 Figure S2 Maximum parsimony consensus tree obtained in the analysis of plastid DNA sequences Maximum parsimony consensus tree obtained in the analysis of plastid DNA sequences (*trnH-psbA*, *trnT-L*, *trnL-F*, *ndhF* and *matk*) from 163 samples representing 152 Pooideae, 6 PACMAD, 3 Bambusoideae and 1 Oryzoideae grass species and * Joinvillea ascendens* as outgroup, constructed with PAUP 4.0 beta 10. Dashed lines represent branches with bootstrap support (BS) < 0.9.

10.7717/peerj.3815/supp-8 Figure S3 Maximum clade credibility tree obtained from BEAST analysis of plastid DNA sequences Maximum clade credibility tree obtained from BEAST analysis of plastid DNA sequences (*trnH-psbA*, *trnT-L*, *trnL-F*, *ndhF* and *matk*) from 163 samples representing 152 Pooideae, 6 PACMAD, 3 Bambusoideae and 1 Oryzoideae grass species and *Joinvillea ascendens* used to root the tree. Divergence times were inferred using a relaxed molecular clock model and a birth-death tree model (see Materials and Methods section for details). Dashed lines represent branches with *PPS* < 0.9. Bars correspond to the 95% High Probability Density (HPD) intervals for nodal ages. Qt., Quaternary; Pl., Pliocene; Pt., Pleistocene.

## Additional Information and Declarations

### DNA Deposition

The following information was supplied regarding the deposition of DNA sequences: All sequences produced in this manuscript are available in GenBank (for accession numbers, see Appendix S1). The sequence alignment is available in FigShare (https://doi.org/10.6084/m9.figshare.5132164.v1).

### Data Availability

The following information was supplied regarding data availability: Pimentel, Manuel; Escudero, Marcial; Sahuquillo, Elvira; Minaya, Miguel; Catalán, Pilar (2017): Pimenteletal_sequence_alignment.nex. figshare.
https://doi.org/10.6084/m9.figshare.5132164.v1.
