# Supplementary material for: Are diversification rates and chromosome evolution in the temperate grasses (Pooideae) associated with major environmental changes in the Oligocene-Miocene?
Source: PeerJ. 2017 Sep 22;5:e3815. doi: 10.7717/peerj.3815 (PMC5611942; doi:10.7717/peerj.3815)
Supplement: Appendix S1 — Systematic and phylogenetic adscriptions follow those proposed by Soreng et al., 2003, Soreng et al., 2007, Soreng et al., 2015, and Torrecilla & Catalán, 2002, Torrecilla, López Rodríguez & Catalán, 2004, Catalán, 2006, Quintanar, Castroviejo & Catalán, 2007, Bouchenak-Khelladi et al., 2008, Inda et al., 2008, Schneider et al., 2009, and Minaya et al., 2017. Herbarium codes of official herbaria follow Index Herbariorum (Thiers, consulted June the 16th 2017). UZ, University of Zaragoza (Spain) Herbarium. An asterisk was added to the sequences that were downloaded from Genbank. [file peerj-05-3815-s001.doc]

**Appendix S1.** List of taxa included in this study. Systematic and phylogenetic adscriptions follow those proposed by Soreng *et al.*, 2003, 2007, 2015, and Torrecilla *et al.*, 2002, 2004, Catalan *et al.*, 2006, Quintanar *et al.*, 2007, Bouchenak-Khelladi *et al.*, 2008, Inda *et al.*, 2008, Schneider *et al.*, 2009, and Minaya *et al*. 2017. Herbarium codes of official herbaria follow *Index Herbariorum* (Thiers, consulted June the 16^th^ 2017). UZ, University of Zaragoza (Spain) Herbarium. An asterisk was added to the sequences that were downloaded from Genbank. All references in this appendix are cited in the main article.

| **Taxa** | **Source** | **GenBank accession** | | | | |
| --- | --- | --- | --- | --- | --- | --- |
|  |  | ***TrnH-PsbA*** | ***mat*K** | ***TrnT-L*** | ***TrnL-F*** | ***ndh*F** |
| **Joinvilleaceae Tomi. and A.C.Sm.** | | | | | | |
| *Joinvillea ascendens* Gaud. ex Brongn & Gris | E. A. Kellogg collection, Genbank | - | AF164380* | JQ972979* | JQ972955* | U21973* |
| **PACMAD clade** | | | | | | |
| **Panicoideae Link. (including Centothecoideae Soderstrom)** | | | | | | |
| *Chasmanthium latifolium* (Michx.) H.O. Yates | USA, cultivated seeds, G. Sanchez-Ken s.n, Genbank | KJ529116 | KJ529308 | KJ529257 | EF137558 | GU359720 |
| *Zea mays* L. | Genbank | GU575286* | X86563* | EF541347* | EF541269* | U21985.1* |
| **Micrairoideae Pilger** | | | | | | |
| *Arundo donax* L. | Genbank | HE966493* | AF164408* | KF169820* | GQ869907* | U21998* |
| *Panicum miliaceum* L. | Genbank | - | FR667662.1* | JQ972982* | JQ972957* | FR667670* |
| **Danthonioideae Barker & Linder** | | | | | | |
| *Danthonia decumbens* (L.) DC | Spain. Los Alcornocales, UZ 31.08, Genbank | KJ529117 | KJ529309 | JQ972986 | EU401189 | KJ529423 |
| **Chloridoideae Kuth ex Beilschm.** | | | | | | |
| *Sporobolus indicus* (L.) R.Br. | Genbank | - | AF144601* | JQ972992* | EF137569* | U21983.1* |
| **BOP clade** | | | | | | |
| **Ehrhartoideae Link** | | | | | | |
| *Oryza sativa* L. | Genbank | GU575284* | AF148650* | DQ415935* | EF137577* | X15901* |
| **Bambusoideae Luerss.** | | | | | | |
| *Arundinaria gigantea* (Walter) Muhl. | Genbank | FJ644249* | - | GQ468376* | EF137522* | JX235347* |
| *Bambusa vulgaris* Schrad. ex J.C. Wendl. | Genbank | FJ644251* | FJ970915* | FJ644133* | JX428443* | HE573559* |
| *Otatea acuminata* (Munro) C.E.Calderon & Soderstr. | Genbank | EF589645* | EF137436.1* | JQ408612* | EF137542* | AF182350* |
| *Pariana radicifolia* Sagot ex Döll | Genbank | FJ644260.1* | AF164387* | FJ644168* | EF137543* | AF182354* |
| **Pooideae Benth.** | | | | | | |
| **Brachyelytreae Ohwi** | | | | | | |
| *Brachyelytrum erectum* (Schreb.)P. Beauv. | USA: Indiana UZ 114.08; Genbank | KJ529118 | KJ529310 | FJ644136* | EU489326* | KJ529424 |
| **Nardeae W.D.J. Koch + Lygeeae J. Presl complex** | |  |  |  |  |  |
| *Nardus stricta* L. | Spain. Granada. UZ 229.07; Genbank | KJ529119 | EU489217* | JQ972994* | EU434097* | JX438109* |
| *Lygeum spartum* L. | Spain. Zaragoza: vedado de Peñaflor, UZ 136.07; Genbank | KJ529120 | KJ529311 | - | EU434098* | JX438110* |
| **Meliceae Endl.** |  |  |  |  |  |  |
| *Glyceria declinata* Bréb. | Spain. Caceres. UZ 25.07; Genbank | KJ529121 | KJ529312 | KJ529258 | EU223364* | KJ529425 |
| *Melica minuta* L. | Spain. Córdoba.UZ 102.07 | KJ529122 | KJ529313 | KJ529259 | KJ529403 | KJ529426 |
| **Stipeae Dumort.** | | | | | | |
| *Achnatherum calamagrostis* (L.) P. Beauv. | Spain. Huesca: Sra. Guara, UZ 282.07; Genbank | KJ529123 | KJ529314 | JQ972996* | EU200806* | JX438111* |
| *Stipa offneri* Breistr. | Spain. Zaragoza. UZ 135.07; Genbank | KJ529124 | KJ529315 | JQ972997* | JQ972961* | JX438112* |
| *Stipa tenacissima* L. | Spain: Almeria. UZ 180.0 | - | KJ529316 | KJ529260 | KJ529404 | KJ529427 |
| **Brachypodieae Harz + Diarrheneae C.S. Campb.** | | | | | | |
| *Diarrhena americana* P. Beauv. | USA. Indiana: Belmont. UZ 116.08; Genbank | KJ529125 | FM253123* | JQ972998* | JQ972962* | KJ529428 |
| *Brachypodium distachyon* (L.) P. Beauv. | Spain. Zaragoza. UZ 120.07; Genbank | KJ529126 | KJ529317 | JQ972999* | AF478500* | KJ529429 |
| *Brachypodium boissieri* Nyman | Spain. Sra. Nevada. UZ 211.07; Genbank | - | JX438065* | JQ973000* | JQ972964* | JX438113* |
| **Triticodae T.D. Macfarl. & L. Watson (Bromeae Dumort. / Triticeae Dumort. Complex)** | | | | | | |
| *Bromus ramosus* Huds. | Spain. León, Macizo Central. LEB WGS84 | KJ529127 | KJ529318 | KJ529261 | KJ529405 | KJ529430 |
| *Bromus rubens* L. | Spain. Almería, Tabernas; Genbank | KJ529128 | KJ529319 | KJ529262 | EU036169* | KJ529431 |
| *Bromus squarrosus* L. | Spain. León. LEB WP13 | KJ529129 | KJ529320 | KJ529263 | KJ529406 | KJ529432 |
| *Bromus tectorum* L. | Spain. León. LEB WP8. Genbank | KJ529130 | KJ529321 | KJ529264 | EU036166* | KJ529433 |
| *Bromus hordeaceus* L. | Spain. León | KJ529131 | KJ529322 | - | KJ529407 | KJ529434 |
| *Triticum aestivum* L. | Genbank | GU575287* | DQ420028* | JQ973001* | AF148757* | KC912694* |
| *Agropyron cristatum* (L.) Gaertn. | Spain. Zaragoza. UZ 134.07; Genbank | KJ529132 | JX438066* | AF519115* | AY740792* | JX438114* |
| *Elymus fartus* (Viv.) Runemark ex Melderis | Spain A Coruña. SANT69537; Genbank | KJ529133 | KF277162* | KJ529265 | - | AM849122* |
| *Dasypyrum villosum* (L.) P. Candargy | Italy. Sardinia. UZ 158.07.  Genbank | KJ529134 | KJ529323 | DQ419995* | JQ972965* | JX438115* |
| *Secale cereale* L. | Spain. León; Genbank | KJ529135 | KJ529324 | DQ336856* | AF478501* | KJ529435 |
| *Hordeum vulgare* L. | Genbank | - | AB078138* | EU036136* | EU036163* | HVU22003* |
| *Aegilops geniculata* Roth | Spain. Zaragoza. UZ 138.07 Genbank | KJ529136 | JX438067* | EU013843* | EU013604* | JX438116* |
| **Poodae L. Liu (Aveneae Dumort. / Poeae R.Br)** | | | | | | |
| **Aveneae core lineages (Aveneae-type plastid DNA)** | | | | | | |
| *Phalaris canariensis* L. | Spain. Valencia. UZ 338.07; Genbank | KJ529137 | JX438068* | DQ631509* | DQ631443* | JX438117* |
| *Phalaris coerulescens* Desf. | Spain: Badajoz. UZ 50.07 | KJ529138 | KJ529325 | KJ529266 | KJ529408 | KJ529436 |
| *Phalaris minor* Retz. | Spain. Zaragoza. UZ 137.07 | KJ529139 | - | KJ529267 | KJ529409 | KJ529437 |
| *Ammophila arenaria* (L.) Link | Spain. Huelva. UZ 75.07; Genbank | KJ529140 | KJ529326 | DQ631522* | DQ631456* | JX438118* |
| *Agrostis capillaris* All. | Spain. Lugo. UZ 299.07; Genbank | KJ529141 | KJ529327 | AY450936* | JQ972966* | JX438119* |
| *Agrostis stolonifera* L. | Spain. Lugo. UZ 300.07;  Genbank | HQ596587* | KJ529328 | KJ529268 | KJ529410 | KJ529438 |
| *Agrostis curtisii* Kerguélen | Spain. Lugo. UZ 314.07 | KJ529142 | KJ529329 | KJ529269 | - | KJ529439 |
| *Neoschischkinia truncatula* (Parl.) Valdés & H. Scholz | Spain. Pontevedra. UZ 311.07 | - | KJ529330 | KJ529270 | - | KJ529440 |
| *Triplachne nitens* Link. | Spain. Almeria UZ 365.07 | KJ529143 | KJ529331 | DQ336861* | DQ336836* | JX438120* |
| *Chaetopogon fasciculatus* (Link.) Hayek. | Spain. Huelva UZ 77.07; Genbank | KJ529144 | - | DQ631523* | DQ631457* | JX438121* |
| *Polypogon maritimus* Willd. | Spain. Badajoz. UZ 52.07; Genbank | KJ529145 | KJ529332 | DQ336863* | DQ336838* | JX438122* |
| *Anthoxanthum aristatum* Boiss. | Spain. Caceres UZ 24.07; Genbank | KJ529146 | KJ529333 | DQ631515* | DQ631449* | JX438123* |
| *Avena sterilis* L. | Spain. Caceres. UZ 19.07;  Genbank | KJ529147 | JX438073* | JQ973002* | EU833883* | JX438124* |
| *Avena barbata* Pott. ex Link. | Spain. Badajoz. UZ 51.07 | KJ529148 | KJ529334 | KJ529271 | KJ529411 | KJ529441 |
| *Helictotrichon filifolium* (Lag.) Henrard | Spain. Valencia. UZ 346.07; Genbank | KJ529149 | - | DQ336864* | DQ336839* | JX438125* |
| *Arrhenatherum elatius* (L.) P. Beauv. ex. Z. Presl. and C. Presl. | Spain. Badajoz. UZ 55.07;  Genbank | FJ395505* | KJ529335 | DQ336866* | EF137591* | KJ529442 |
| *Pseudarrhenatheum longifolium* Rouy. | Spain. Coruña UZ 298.07;  Genbank | KJ529150 | JX438075* | JQ973003* | JQ972967* | JX438127* |
| *Lagurus ovatus* L. | Spain. Huelva. UZ 71.07; Genbank | KJ529151 | KJ529336 | DQ631530* | DQ631464* | KJ529443 |
| *Gaudinia fragilis* (L.) P. Beauv. | Spain. Caceres. UZ 30.07;  Genbank | KJ529152 | EF137499* | DQ631545* | DQ631478* | KJ529444 |
| *Trisetum loeflingianum* (L.) C. Presl. | Spain. Toledo. UZ 10.07; Genbank | KJ529153 | JX438076* | DQ631539* | DQ631473* | JX438128* |
| *Koeleria vallesiana* (Honck.) Gaudin | Spain. Madrid. UZ 4.07; Genbank | KJ529154 | JX438077* | DQ631536* | DQ631468* | JX438129* |
| *Koeleria castellana* Boiss. & Reut. | Spain. Madrid. UZ 16.07 | KJ529155 | KJ529337 | KJ529272 | - | KJ529445 |
| *Koeleria crassipes* Lange. | Spain. Madrid. UZ 7.07;  Genbank | KJ529156 | KJ529338 | KJ529273 | DQ631535* | KJ529446 |
| *Parafestuca albida* (Lowe) E.B. Alexeev | Portugal. Madeira; Genbank | - | DQ786930* | DQ336869* | AF533022* | DQ786858* |
| *Rostraria cristata* (L.) Tzvelev | Spain. Toledo. UZ 9.07; Genbank | KJ529157 | JX438078* | DQ336879* | DQ336853* | JX438130* |
| *Rostraria salzmannii* (Boiss. & Reut.) Holub | Spain. Castellón. UZ329.07 | KJ529158 | KJ529339 | KJ529274 | KJ529412 | KJ529447 |
| *Avellinia michelii* (Savi) Parl. | Spain. Cádiz. UZ 98.07; Genbank | KJ529159 | KJ529340 | DQ631531* | DQ631465* | KJ529448 |
| **Poeae pro parte plus former Aveneae pro parte (Poeae-type plastid DNA)** | | | | | | |
| *Molineriella laevis* (Brot.) Rouy | Spain. Toledo UZ 17.07;  Genbank | KJ529160 | DQ786929* | KJ529275 | KJ529413 | KJ529449 |
| *Holcus lanatus* L. | Spain. Leon. UZ 291.07; Genbank | KJ529161 | KJ529341 | DQ631503* | DQ631437* | KJ529450 |
| *Poa bulbosa* L. | Spain. Alicante. UZ 334.07; Genbank | KJ529162 | KJ529342 | EU792472* | DQ354035* | KJ529451 |
| *Poa infirma* Kunth. | Spain. Caceres. UZ 37.07; Genbank | KJ529163 | KJ529343 | DQ367407* | AF488773* | KJ529452 |
| *Poa annua* L. | Spain. León. WP16 LEB.; Genbank | KJ529164 | KJ529344 | DQ353983* | FJ490809* | KJ529453 |
| *Poa ligulata* Boiss. | Spain. Toledo. UZ 12.07 | KJ529165 | KJ529345 | KJ529276 | - | KJ529454 |
| *Poa pratensis* L. | Spain. Leon. WP14. LEB | KJ529166 | KJ529346 | KJ529277 | - | KJ529455 |
| *Poa compressa* L. | Spain. Lugo; Genbank | KJ529167 | KJ529347 | DQ354003* | AY504649* | KJ529456 |
| *Arctagrostis latifolia* (R. Br.) Griseb. | Genbank | - | DQ786885* | DQ353969* | DQ353969* | DQ786813* |
| *Desmazeria marina* (L.) Druce. | Spain. Alicante. UZ 320.07 | KJ529168 | KJ529348 | KJ529278 | KJ529414 | KJ529457 |
| *Catapodium rigidum* (L.) C.E. Hubb. | Spain. Zaragoza. UZ 124.07; Genbank | KJ529169 | JX438086* | JQ973007* | - | JX438141* |
| *Desmazeria sicula* (Jacq.) Dumort. | Genbank | - | HE646576* | EF584989* | EF592948* | DQ786832* |
| *Alopecurus arundinaceus* Poir. | Spain. Badajoz. UZ 53.07; Genbank | KJ529170 | JX438080* | JQ973004* | JQ972968* | JX438133* |
| *Catabrosa aquatica* (L.) P. Beauv. | Spain. Huesca; Genbank | KJ529171 | JX438061* | JQ973005* | JQ972969* | JX438135* |
| *Puccinellia distans* (Jacq.) Parl. | Spain. Barcelona. UZ156.07; Genbank | KJ529172 | KJ529349 | DQ336859* | AF533024* | JX438134* |
| *Aira cupaniana* Guss. | Spain. Caceres.UZ 21.07; Genbank | - | KJ529350 | DQ631508* | DQ631442* | KJ529458 |
| *Corynephorus canescens* (L.) P. Beauv. | Spain. Huelva. UZ 93.07; Genbank | - | KJ529351 | DQ631506* | - | JX438136* |
| *Deschampsia cespitosa* (L.) P. Beauv. | Spain. Lugo. UZ 305.07; Genbank | FJ395500* | KJ529352 | EF584984* | AF533026* | KJ529459 |
| *Avenella flexuosa* (L.) Drejer | Spain. Lugo. UZ 313.07; Genbank | KJ529173 | JX438083* | DQ631505* | AY237913* | JX438137* |
| *Periballia involucrata* Jonka | Spain. Salamanca. UZ 364.07; Genbank | KJ529174 | KJ529353 | DQ631504* | DQ631438* | KJ529460 |
| *Airopsis tenella* Coss. & Durand | Spain. Huelva. UZ 76.07; Genbank | KJ529175 | KJ529354 | KJ529279 | DQ631445* | KJ529461 |
| *Helictotrichon sulcatum* (J. Gay ex Boiss.) Potztal | Spain. Caceres. UZ 33.07; Genbank | KJ529176 | KJ529355 | DQ631527* | DQ631461* | KJ529462 |
| *Helictochloa marginata* (Lowe) Romero Zarco | Spain. Córdoba. UZ 101.07; Genbank | - | KJ529356 | KJ529280 | DQ631459* | - |
| *Mibora minima* (L.) Desv. | Spain. Toledo. UZ 14.07; Genbank | - | KJ529357 | DQ631520* | DQ631454* | DQ786855* |
| *Briza minor* L. | Spain. Caceres, UZ 39.07; Genbank | KJ529177 | KJ529358 | KJ529281 | EU395903* | KJ529463 |
| *Briza maxima* L. | Spain. Lugo; Genbank | KJ529178 | KJ529359 | - | EU395901* | HE575736* |
| *Antinoria agrostidea* (DC.) Parl.. | Spain. Lugo. UZ 297.07 | KJ529179 | KJ529360 | - | - | KJ529464 |
| *Echinaria capitata* (L.) Desf. | Spain. Toledo. UZ 13.07; Genbank | KJ529180 | KJ529361 | DQ631519* | DQ631453* | JX438138* |
| *Cynosurus echinatus* L. | Spain. Lugo. UZ 295.07; Genbank | KJ529181 | KJ529362 | EF584993* | AF533031* | KJ529465 |
| *Cynosurus cristatus* L. | Spain. Leon. UZ 285.07; Genbank | - | KJ529363 | JQ973006* | EF137599* | JX438139* |
| *Parapholis incurva* (L.) C.E. Hubb. | Spain. Zaragoza. UZ 127.07; Genbank | KJ529182 | KJ529364 | EF584991* | AF533036* | KJ529466 |
| *Parapholis filiformis* (Roth) C.E. Hubb. | Spain. Castellón. UZ 326.07; Genbank | KJ529183 | KJ529365 | KJ529282 | KJ529415 | KJ529467 |
| *Hainardia cylindrica* (Willd.) Greuter | Spain. Alicante. UZ 356.07; Genbank | KJ529184 | KJ529366 | EF584990* | AF533035* | KJ529468 |
| *Dactylis hispanica* Roth | Spain. Zaragoza. UZ 116.07; Genbank | KJ529185 | KJ529367 | EF584994* | AF533027* | KJ529469 |
| *Dactylis glomerata* L. | Spain. Caceres. UZ 27.07; Genbank | KJ529186 | KJ529368 | KJ529283 | AY327794* | KJ529470 |
| *Dactylis juncinella* Bory | Spain. Granada. UZ 222.07; Genbank | - | KJ529369 | - | KJ529416 | KJ529471 |
| *Lamarckia aurea* (L.) Moench | Spain. Caceres. UZ 36.07; Genbank | KJ529187 | JX438085* | EF584995* | AF533029* | JX438140* |
| *Cutandia maritima* (L.) Barbey | Spain. Valencia. UZ 324.07; Genbank | KJ529188 | KJ529370 | EF584987* | AF487618* | KJ529472 |
| **Loliinae Dumort.** | | | | | | |
| **Broad-Leaved Loliinae** | | | | | | |
| *Festuca modesta* Nees ex Steud. | China. Yunnan. US-3420887; Genbank | KJ529189 | KJ529371 | EF585068* | EF592985* | JX438143* |
| *Festuca triflora* J.F. Gmel. | Spain. Cádiz. UZ 95.2000; Genbank | KJ529190 | JX438088* | EF585109* | AF533052* | JX438144* |
| *Festuca coerulescens* Desf. | Spain. Cádiz. UZ 19.08; Genbank | KJ529191 | JX438089* | EF585027* | JQ972971* | JX438145* |
| *Festuca pseudoeskia* Boiss. | Spain. Granada. UZ 73.2000; Genbank | KJ529192 | JX438090* | EF585084* | AY099000* | JX438146* |
| *Festuca altissima* All. | Norway. Akershus; Genbank | KJ529193 | KJ529372 | KJ529284 | AF478505* | - |
| *Festuca scariosa* (Lag.) Asch. & Graebn. | Spain. Granada. UZ 219.07; Genbank | KJ529194 | KJ529373 | EF585100* | AY098999* | JX438148* |
| *Festuca lasto* Boiss. | Spain. Cádiz. UZ 146.07; Genbank | KJ529195 | KJ529374 | EF585060* | AY098998* | KJ529473 |
| *Festuca drymeja* Mert. & W.D.J. Koch | Hungary. Balaton LEI; Genbank | KJ529196 | KJ529375 | EF585034* | AY098997* | KJ529474 |
| *Festuca donax* Lowe | Portugal. Madeira. MS4515; Genbank | - | KJ529376 | EF585033* | EF592968* | KJ529475 |
| *Festuca kingii* Scribn. | USA. Colorado. PC 1.93. UZ; Genbank | KJ529197 | KJ529377 | EF585058* | AY099004* | DQ786851* |
| *Festuca paniculata* (L.) Schinz & Thell. | Spain. León. UZ 290.07 Genbank | KJ529198 | JX438091.1* | JQ973008* | AF533046 | JX438149* |
| *Festuca fenas* Lag. | France. BN354. UZ 142.07; Genbank | KJ529199 | JX438092* | JQ973009* | JQ972973* | JX438150* |
| *Festuca mairei* St.-Yves | Morocco. Marrakech. 4064. UAM; Genbank | KJ529200 | KJ529378 | KJ529285 | AY098996* | KJ529476 |
| *Festuca arundinacea* Schreb. | Spain: Lugo. José Ángel López Rodríguez (JALR) 1081; Genbank | KJ529201 | JX438093* | DQ367405* | AF533042* | JX438151* |
| *Micropyropsis tuberosa* Romero-Zarco & Cabezudo | Spain. Huelva. UZ 89.07; Genbank | KJ529202 | KJ529379 | JQ973010* | AF533037* | JX438152* |
| *Festuca pratensis* Huds. | Spain. Huesca. Jaca. JACA 544795; Genbank | KJ529203 | JX438095* | JQ973011* | AF478503* | JX438153* |
| *Festuca gigantea* (L.) Vill. | Norway; Genbank | KJ529204 | JX438096* | JQ973012* | AF533043* | JX438154* |
| *Lolium canariense* Steud. | Cape Verde. LPA 21.399; Genbank | - | KJ529380 | EF379014* | AY228162* | KJ529477 |
| *Lolium rigidum* Gaudin | Spain. Zaragoza. UZ 18.2000; Genbank | KJ529205 | KJ529381 | KJ529286 | EF378984* | KJ529478 |
| *Lolium perenne* L. | United Kingdom; Genbank | KJ529206 | JX438097* | EF379024* | EF378973* | JX438155* |
| *Lolium multiflorum* Lam. | Spain. Alicante UZ 332.07; Genbank | KJ529207 | KJ529382 | KJ529287 | EF378972* | KJ529479 |
| *Lolium persicum* Boiss. & Hohen. | Turkey. Cultivated seeds USDA Plant Introduction (PI) 545661; Genbank | KJ529208 | KJ529383 | EF379027* | EF378975* | KJ529480 |
| *Lolium remotum* Schrank | Sweden. Cultivated seeds PI 233613; Genbank | KJ529209 | - | - | EF378979* | KJ529481 |
| *Festuca font-queri* St.-Yves | Morocco. Rif Mountains; Genbank | KJ529210 | - | KJ529288 | AF533044* | KJ529482 |
| *Festuca africana* (Hack.) Clayton | Uganda. Elgon Mnt. MHU1585; Genbank | KJ529212 | - | KJ529290 | KJ529418 | KJ529483 |
| *Festuca baetica* (Hack.) Richt. | Spain. Granada. UZ 205.07; Genbank | KJ529211 | KJ529384 | KJ529289 | KJ529417 | KJ529484 |
| **Fine-Leaved Loliinae** | | | | | | |
| *Festuca simensis* Hochst. ex A. Rich. | Uganda. Echuya., MHU 1608; Genbank | KJ529213 | - | KJ529291 | GU573750* | KJ529485 |
| *Vulpia geniculata* (L.) Link | Spain. Sevilla. J29397; Genbank | KJ529214 | - | KJ529292 | AF478531* | - |
| *Festuca elegans* Boiss. | Spain. Granada. UZ 231.07; Genbank | KJ529215 | KJ529385 | EF585038* | EF585038* | KJ529486 |
| *Festuca gautieri* (Hack.) K. Richt. | Spain. Granada. UZ 232.07; Genbank | KJ529216 | JX438099* | EF585044* | AF478507* | JX438157* |
| *Festuca eskia* Ramond ex DC. | Spain. Huesca; Genbank | KJ529217 | JX438100* | EF585040* | AF478508* | JX438158* |
| *Vulpia ciliata* Dumort. | Spain. Zaragoza. UZ 112.07; Genbank | KJ529218 | KJ529386 | EF585120* | AF478534* | KJ529487 |
| *Psilurus incurvus* (Gouan) Schinz & Thell. | Spain. Caceres UZ 31.07; Genbank | KJ529219 | JX438059* | JQ973013* | AF478533* | JX438159* |
| *Narduroides salzmannii* (Boiss.) Rouy | Spain. Madrid. UZ 111.07 Genbank | KJ529220 | JX438101.1* | JQ973014* | AF478535* | JX438160* |
| *Micropyrum tenellum* (L.) Link | Spain. Badajoz. UZ 46.07; Genbank | KJ529221 | KJ529387 | EF585116* | AF478534* | KJ529488 |
| *Festuca capillifolia* Dufour ex Roem. & Schult. | Spain. Almeria. UZ 179.07; Genbank | KJ529222 | JX438102* | EF585022* | AF478511* | JX438161* |
| *Festuca clementei* Boiss. | Spain. Granada. UZ 223.07; Genbank | KJ529223 | JX438060* | EF585025* | AF478524* | JX438162* |
| *Festuca borderei* Kerguélen | Spain. Huesca; Genbank | KJ529224 | - | KJ529293 | AF478510* | KJ529489 |
| *Festuca plicata* Hack. | Spain. Granada. UZ 204.07; Genbank | KJ529225 | - | KJ529294 | AF478525* | KJ529490 |
| *Vulpia unilateralis* (L.) Stace | Spain. Zaragoza.UZ 114.07; Genbank | KJ529226 | KJ529388 | EF585130* | AY118106* | KJ529491 |
| *Vulpia membranacea* (L.) Dumort. | Spain. Cádiz. UZ 97.07; Genbank | KJ529227 | - | EF585124* | AY118101* | KJ529492 |
| *Vulpia fasciculata* (Forssk.) Samp | Spain. Barcelona. UZ 15.2000; Genbank | KJ529228 | - | EF585121* | AF478528* | JX438163* |
| *Wangenheimia lima* (L.) Trin. | Spain. Zaragoza.UZ 113.07; Genbank | KJ529229 | JX438103* | EF585131* | AF478536* | JX438165* |
| *Festuca ovina* L. | Germany. Thüringen; Genbank | KJ529230 | JX438104* | EF585076* | JQ972975* | JX438166* |
| *Festuca indigesta* Boiss. | Spain. Granada. UZ 212.07; Genbank | KJ529231 | JX438105* | EF585054* | AF478519* | JX438167* |
| *Festuca hystrix* Boiss. | Spain. Almeria. UZ 185.07; Genbank | KJ529232 | KJ529389 | EF585051* | AF478520* | JX438168* |
| *Festuca longiauriculata* Fuente, Ortúñez & Ferrero | Spain. Almería. UZ 59.2000; Genbank | KJ529233 | KJ529390 | EF585062* | AF478518* | KJ529493 |
| *Festuca frigida* Grossh. | Spain. Granada. UZ 226.07; Genbank | KJ529234 | - | DQ631485* | AF478521* | KJ529494 |
| *Festuca fragilis* (Luces) B. Briceño | Spain. Mérida; Genbank. | KJ529235 | - | KJ529295 | EF593014* | KJ529495 |
| *Vulpia muralis* (Kunth) Nees | Spain. Sevilla. PC 1.2002; Genbank | KJ529236 | KJ529391 | EF585126* | AY118102* | KJ529496 |
| *Vulpia bromoides* (L.) Gray | Spain. Lugo.. JALR 01080; Genbank | KJ529237 | KJ529392 | KJ529296 | AY528936* | KJ529497 |
| *Vulpia alopecuros* (Schousb.) Dumort. | Portugal. Algarve. LEI; Genbank | KJ529238 | KJ529393 | EF585117* | AF487617* | KJ529498 |
| *Vulpia fontquerana* Melderis & Stace | Spain. Huelva. UZ 91.07 | KJ529239 | - | KJ529297 | KJ529419 | KJ529499 |
| *Vulpia microstachys* (Nutt.) Munro | USA. California. Soreng R. 7406; Genbank | KJ529240 | KJ529394 | KJ529298 | EF593015* | KJ529500 |
| *Vulpia octoflora* (Walter) Rydb. | USA. Washington.Peterson P.M. 3263; Genbank | KJ529241 | - | KJ529299 | EF593016* | KJ529501 |
| *Festuca rivularis* Boiss. | Spain. Granada. UZ 78.2000; Genbank | KJ529242 | KJ529395 | EF585093* | AF478512* | KJ529502 |
| *Festuca jubata* Lowe | Portugal. Madeira;.Genbank | KJ529243 | - | KJ529300 | EF592980* | KJ529503 |
| *Festuca agustinii* Linding. | Spain. Canarias; Genbank | KJ529244 | KJ529396 | EF585000* | EF592949* | KJ529504 |
| *Festuca francoi* Fern. Prieto, C. Aguiar, E.Dias & M.I.Gut. | Portugal. Azores. Sequeira M. 4403 | KJ529245 | - | KJ529301 | KJ529420 | KJ529505 |
| *Festuca rubra* L. | UK. Scotland.UZ 150.07; Genbank | KJ529246 | JX438106* | EF585097* | EF593001* | JX438169* |
| *Festuca iberica* (Hack.) K. Richt. | Spain. Granada.UZ 218.07; Genbank | - | KJ529397 | EF585052* | AF478516* | KJ529506 |
| *Festuca ampla* Hack. | Spain. Huelva; Genbank | KJ529247 | - | EF585007* | EF592953* | KJ529507 |
| *Festuca rothmaleri* (Litard.) Markgr.-Dann. | Spain. Madrid; Genbank | KJ529248 | - | KJ529302 | AF478513* | KJ529508 |
| *Festuca nevadensis* (Hack.) K. Richt. | Spain. Granada., UZ 202.07; Genbank | KJ529249 | KJ529398 | EF585071* | AF478514* | KJ529509 |
| *Festuca abyssinica* Hochst. ex A. Rich. | Uganda. Gabinga. MHU1604; Genbank | KJ529250 | JX438107* | JQ973016* | JQ972976* | JX438171* |
| *Festuca pulchella* Schrad. | Switzerland. Bern. JM 7807; Genbank | KJ529251 | KJ529399 | EF585086* | AF519985* | KJ529510 |
| *Festuca dichoclada* Pilg. | Peru. Junin. US-3421417;  Genbank | KJ529252 | - | KJ529303 | EF592967* | KJ529511 |
| *Sesleria albicans* Kit. Ex Schult. | Spain. León | KJ529253 | KJ529400 | KJ529304 | KJ529421 | KJ529512 |
| *Festuca argentea* Savi | Spain. Burgos; Genbank | KJ529254 | KJ529401 | KJ529305 | AF533030* | KJ529513 |
| *Oreochloa confusa* (Coincy) Rouy | Spain. Cantabria | KJ529255 | KJ529402 | KJ529306 | KJ529422 | KJ529514 |
| *Festuca alpina* Suter | Spain. Huesca; Genbank | KJ529256 | - | KJ529307 | AF478522* | KJ529515 |
