# Supplementary material for: Are diversification rates and chromosome evolution in the temperate grasses (Pooideae) associated with major environmental changes in the Oligocene-Miocene?
Source: PeerJ. 2017 Sep 22;5:e3815. doi: 10.7717/peerj.3815 (PMC5611942; doi:10.7717/peerj.3815)
Supplement: Appendix S2 — Procedures for DNA isolation, DNA amplification and sequencing, and for sequence alignment. [file peerj-05-3815-s002.doc]

**Appendix S2. Expanded materials and methods. Procedures for DNA isolation, DNA amplification and sequencing, and for sequence alignment**

DNA from fresh or silica gel-dried field-collected leaves was extracted following the 2% CTAB procedure of Doyle and Doyle (1990). DNA from herbarium samples was extracted using the NucleoSpin Plant II kit (Macherey-Nagel, Düren, Germany) following the manufacturer’s protocol.

The *trnH*-*psbA* intergenic spacer was amplified using the primers of Tate and Simpson (2003) and Sang *et al.* (1997) following Shaw *et al.*’s (2005) procedure. The *trnT-L* and *trnL-F* regions were amplified using the primers a and b and c and f, respectively (Taberlet *et al.*, 1991). Amplification of the *trnL-F* region was conducted following Torrecilla *et al.* (2003), whereas the *trnT-L* region was amplified following Galley and Linder (2007). Amplification of the plastid coding regions *matK* and *ndhF* was achieved following Schneider *et al.* (2009) and Clarke *et al.* (1995), respectively.

Amplified products were purified using ExoSap-IT PCR cleanup reagent (Affymetrix, Santa Clara, California). Products were sequenced using the BigDye Terminator Cycle Sequencing Ready Reaction v3.1 kit (Applied Biosystems, Paisley, United Kingdom) on an Applied Biosystems 3710 automated sequencer (Applied Biosystems, Paisley, United Kingdom). Amplification primers were also used for sequencing in all cases except for the *matK* region (Schneider *et al.*, 2009).

The forward and reverse electropherograms were assembled and edited using CodonCode Aligner v. 4.0 (CodonCode Corporation, Dedham, Massachusetts). All DNA regions were aligned separately using the MUSCLE algorithm (Edgar, 2004) as implemented in the software SeaView v. 4.0 (Gouy *et al.*, 2010) and manually adjusted when errors were detected. For the alignment of coding regions, the aminoacid sequence was used.

LITERATURE CITED

**Clarke L, Zhang GW, Wendel JF. 1995.** A phylogeny of the grass family (Poaceae) based on *ndhF* sequence data. *Systematic Botany* **20**: 436-460.

**Doyle JJ, Doyle JL. 1990.** Isolation of plant DNA from fresh tissue. *Focus* **12**: 13-15.

**Edgar RC. 2004.** MUSCLE: multiple sequence alignment with high accuracy and high throughput. *Nucleic Acids Research* **32**: 1792–1797.

**Galley CA, Linder HP. 2007.** The phylogeny of the *Pentaschistis* clade (Danthonioideae, Poaceae) based on chloroplast DNA, and the evolution and loss of complex characters. *Evolution* **61**: 864–884.

**Gouy M, Guindon S, Gascuel O. 2010.** SeaView version 4: a multiplatform graphical user interface for sequence alignment and phylogenetic tree building. *Molecular Biology and Evolution* **27**: 221–224.

**Sang T, Crawford DJ, Stuessy TF. 1997.** Chloroplast DNA phylogeny, reticulate evolution and biogeography of *Paeonia* (Paeoniaceae). *American Journal of Botany* **84**: 1120–1136.

**Schneider J, Doring E, Hilu KW, Roser M. 2009.** Phylogenetic structure of the grass subfamily Pooideae based on comparison of plastid matK gene-3’ trnK exon and nuclear ITS sequences. *Taxon* **58**: 405–424.

**Shaw J, Lickey EB, Beck JT, Farmer SB, Liu W, Miller J, Siripun, Winder CT, Schilling EE, Small RL. 2005.** The tortoise and the hare II. Relative utility of 21 noncoding chloroplast DNA sequences for phylogenetic analysis. *American Journal of Botany* **92**:142-166.

**Taberlet P, Gielly G, Pautou G, Bouvet J. 1991.** Universal primers for amplification of three non-coding regions of chloroplast DNA. *Plant Molecular Biology* **17**: 1105–1109.

**Tate JA, Simpson BB. 2003.** Paraphyly of *Tarasa* (Malvaceae) and diverse origins of the polyploid species. *Systematic Botany* **28**: 723–737.

**Torrecilla P, López-Rodríguez JA, Stancik D, Catalan P. 2003.** Systematics of *Festuca* sects. *Eskia* Willk., *Pseudatropis* Kriv., *Amphigenes* (Janka) Tzvel., *Pseudoscariosa* Kriv., and *Scariosae* Hack. based on analysis of morphological characters and DNA sequences. *Plant Systematics and Evolution* **239**: 113–139.
