# Supplementary material for: Are diversification rates and chromosome evolution in the temperate grasses (Pooideae) associated with major environmental changes in the Oligocene-Miocene?
Source: PeerJ. 2017 Sep 22;5:e3815. doi: 10.7717/peerj.3815 (PMC5611942; doi:10.7717/peerj.3815)
Supplement: Table S1 — Number of samples sequenced per region (one sample per species); size of the alignment; percentage of parsimony informative characters (p.i.); consistency index (CI) and retention index (RI) (excluding non informative characters). [file peerj-05-3815-s003.doc]

**Table S1.** Number of samples sequenced per region (one sample per species); size of the alignment; percentage of parsimony informative characters (p.i.); consistency index (CI) and retention index (RI) (excluding non informative characters).

| **DNA loci** | **No. of seq. per DNA region** | **Size (bp)** | **Percentage of p. i. characters** | **CI/RI** |
| --- | --- | --- | --- | --- |
| *ndhF* | 168 | 692 | 39.4 | 0.375/0.823 |
| *matk* | 149 | 986 | 31 | 0.434/0.832 |
| *trnH-psbA* | 154 | 581 | 19.7 | 0.420/0.818 |
| *trnL-F* | 166 | 1209 | 18.7 | 0.486/0.810 |
| *trnT-L* | 161 | 1078 | 33.4 | 0.451/0.760 |
| TOTAL | 798 | 4546 | 26 (1505) |  |
