# Supplementary material for: Are diversification rates and chromosome evolution in the temperate grasses (Pooideae) associated with major environmental changes in the Oligocene-Miocene?
Source: PeerJ. 2017 Sep 22;5:e3815. doi: 10.7717/peerj.3815 (PMC5611942; doi:10.7717/peerj.3815)
Supplement: Table S2 — Haploid chromosome number for species included in the study based on: Catalan et al., 1997, Catalán et al., 2004, Díaz-Pérez et al., 2014, Döring et al., 2007, Escobar et al., 2011, Essi et al., 2008, Fortune et al., 2008, Gillespie et al., 2005, Gillespie et al., 2008, Goldblatt & Johnson, 1979, Inda et al., 2013, Kellogg et al., 2015, Minaya et al., 2015, Peterson et al., 2006, Pimentel et al., 2013, Quintanar, Castroviejo & Catalán, 2007, Quintanar et al., 2010, Saarela et al., 2010, Saarela et al., 2015, Schneider et al., 2011, Schneider et al., 2012, Soreng et al., 2007, Soreng et al., 2010, Soreng et al., 2015, Voshell et al., 2011, Watson & Dallwitz, 1992, Winterfeld, Perner & Röser, 2011, Winterfeld et al., 2014. Hp., haploid. [file peerj-05-3815-s004.docx]

**Tabla S2.** Haploid chromosome number for species included in the study based on: Catalan *et al.,* 1997, 2004; Díaz-Pérez *et al*., 2014; Döring *et al*., 2007; Escobar *et al*., 2011; Essi *et al*., 2008; Fortune *et al*., 2008; Gillespie *et al*., 2005, 2008; Goldblatt & Johnson, 1979; Inda *et al*., 2013; Kellogg *et al*., 2015; Minaya *et al*., 2015; Peterson *et al*., 2006, Pimentel *et al*., 2013; Quintanar *et al*., 2007,2010; Saarela *et al*., 2010, 2015; Schneider *et al*., 2011, 2012; Soreng *et al*., 2007, 2010, 2015; Voshell *et al*., 2011; Watson & Dallwitz, 1992; Winterfeld *et al*., 2011, 2014. Hp., haploid.

| **Species** | **Hp. chromosome number** | **Species** | **Hp. chromosome number** |
| --- | --- | --- | --- |
| *Aegilops geniculata* | 7 | *Festuca ovina* | 7 |
| *Agropyron cristatum* | 7 | *Festuca paniculata* | 7 |
| *Agrostis capillaris* | 7 | *Festuca plicata* | 7 |
| *Agrostis curtisii* | 7 | *Festuca pratensis* | 7 |
| *Agrostis stolonifera* | 7 | *Festuca rivularis* | 7 |
| *Aira cupaniana* | 7 | *Festuca rothmaleri* | 7 |
| *Airopsis tenella* | 4 | *Festuca rubra* | 7 |
| *Alopecurus arundinaceus* | 7 | *Festuca scariosa* | 7 |
| *Ammophila arenaria* | 14 | *Festuca simensis* | 7 |
| *Anthoxanthum aristatum* | 5 | *Festuca triflora* | 7 |
| *Antinoria agrostidea* | 7 | *Gaudinia fragilis* | 7 |
| *Arctagrostis latifolia* | 14 | *Hainardia cylindrica* | 13=0.5_7=0.5 |
| *Arrhenatherum elatius* | 7 | *Helictotrichon filifolium* | 7 |
| *Avellinia michelii* | 7 | *Holcus lanatus* | 7 |
| *Avena barbata* | 7 | *Hordeum vulgare* | 7 |
| *Avena sterilis* | 7 | *Koeleria vallesiana* | 7 |
| *Avenula sulcata* | 7 | *Lagurus ovatus* | 7 |
| *Briza minor* | 5=0.5_7=0.5 | *Lamarckia aurea* | 7 |
| *Bromus hordeaceus* | 7 | *Lolium canariense* | 7 |
| *Bromus ramosus* | 7 | *Lolium multiflorum* | 7 |
| *Bromus rubens* | 7 | *Lolium perenne* | 7 |
| *Bromus squarrosus* | 7 | *Lolium persicum* | 7 |
| *Bromus tectorum* | 7 | *Lolium remotum* | 7 |
| *Catabrosa aquatica* | 5 | *Lolium rigidum* | 7 |
| *Chaetopogon fasciculatus* | 7 | *Mibora minima* | 7 |
| *Corynephorus canescens* | 7 | *Micropyropsis tuberosa* | 7 |
| *Cutandia maritima* | 7 | *Micropyrum tenellum* | 7 |
| *Cynosurus cristatus* | 7 | *Molineriella laevis* | 4 |
| *Cynosurus_echinatus* | 7 | *Narduroides salzmannii* | 7 |
| *Dactylis glomerata* | 7 | *Parapholis filiformis* | 7 |
| *Dactylis hispanica* | 7 | *Parapholis incurva* | 7=0.33_9=0.34_19=0.33 |
| *Dasypyrum villosum* | 7 | *Periballia involucrata* | 7=0.5_4=0.5 |
| *Deschampsia cespitosa* | 7=0.5_13=0.5 | *Phalaris canariensis* | 6=0.5_7=0.5 |
| *Deschampsia flexuosa* | 7=0.5_13=0.5 | *Phalaris coerulescens* | 7=0.5_6=0.5 |
| *Desmazeria rigida* | 7 | *Phalaris minor* | 6=0.5_7=0.5 |
| *Echinaria capitata* | 7=0.5_9=0.5 | *Poa annua* | 7 |
| *Elymus sp* | 7 | *Poa bulbosa* | 7 |
| *Festuca abyssinica* | 7 | *Poa compressa* | 7 |
| *Festuca alpina* | 7 | *Poa infirma* | 7 |
| *Festuca ampla* | 7 | *Poa pratensis* | 7 |
| *Festuca arundinacea* | 7 | *Polypogon maritimus* | 7 |
| *Festuca borderei* | 7 | *Psilurus incurvus* | 7 |
| *Festuca capillifolia* | 7 | *Puccinellia distans* | 7 |
| *Festuca donax* | 7 | *Rostraria cristata* | 7 |
| *Festuca drymeja* | 7 | *Secale cereale* | 7 |
| *Festuca elegans* | 7 | *Sesleria albicans* | 7 |
| *Festuca eskia* | 7 | *Triplachne nitens* | 7 |
| *Festuca frigida* | 7 | *Triticum aestivum* | 7 |
| *Festuca gautieri* | 7 | *Vulpia alopecuros* | 7 |
| *Festuca gigantea* | 7 | *Vulpia bromoides* | 7 |
| *Festuca hystrix* | 7 | *Vulpia ciliata* | 7 |
| *Festuca iberica* | 7 | *Vulpia fasciculata* | 7 |
| *Festuca indigesta* | 7 | *Vulpia fontqueriana* | 7 |
| *Festuca lasto* | 7 | *Vulpia membranacea* | 7 |
| *Festuca longiauriculata* | 7 | *Vulpia muralis* | 7 |
| *Festuca mairei* | 7 | *Vulpia octoflora* | 7 |
| *Festuca modesta* | 7 | *Vulpia unilateralis* | 7 |
| *Festuca nevadensis* | 7 | *Wangenheimia lima* | 7 |

**LITERATURA CITED**

**Catalán P, Kellogg EA, Olmstead RG. 1997.** Phylogeny of Poaceae subfamily based on chloroplast *ndhF* gene sequences. *Molecular Phylogenetics and Evolution* **8:** 150–166.

**Catalán P, Torrecilla P, López Rodríguez JA, Olmstead RG. 2004.** Phylogeny of the festucoid grasses of subtribe Loliinae and allies (Poeae, Pooideae) inferred from ITS and *trnL–F* sequences. *Molecular Phylogenetics and Evolution* **31:** 517–541.

**Díaz-Pérez AJ, Sharifi-Tehrani M, Inda LA, Catalán P. 2014.** Polyphyly, gene-duplication and extensive allopolyploidy framed the evolution of the ephemeral *Vulpia* grasses and other fine-leaved Loliinae (Poaceae). *Molecular Phylogenetics and Evolution* **79:** 92–105.

**Döring E, Schneider J, Hilu KW, Röser M. 2007.** Phylogenetic relationships in the Avenae/Poeae complex Pooideae/Poaceae*. Kew Bulettin* **62:** 407-424*.*

**Escobar JS, Scornavacca C, Cenci A, Guilhaumon C, Santoni S, Douzery EJP, Ranwez V, Glémin S, David J. 2011.** Multigenic phylogeny and analysis of tree incongruences in Triticeae (Poaceae). BMC Evolutionary Biology **11:**181

**Essi L, Longhi-Wagner HM, de Souza-Chies TT. 2008.** Phylogenetic analysis of the *Briza* complex (Poaceae). *Molecular Phylogenetics and Evolution* **47:** 1018‑1029.

**Fortune PM, Pourtau N, Viron N, Ainouche ML. 2008**. Molecular phylogeny and reticulate origins of the polyploid *Bromus* species from section *Genea* (Poaceae). *American Journal of Botany* **95(4):** 454–464.

**Gillespie LJ, Soreng RJ. 2005.** A phylogenetic analysis of the bluegrass genus *Poa* based on cpDNA restriction site data. *Systematic Botany* **30:** 84-105.

**Gillespie LJ, Soreng RJ, Bull RD, Jacobs SWL, Refulio-Rodriguez NF. 2008.** Phylogenetic relationships in subtribe Poinae (Poaceae, Poeae) based on nuclear ITS and plastid *trnT-trnL-trnf* sequences. *Botany* **86:** 938-967.

**Goldblatt P, Johnson DE. 1979***.* *Index to Plant Chromosome Numbers (IPCN).* Saint Louis: Missouri Botanical Garden.

**Inda LA, Sanmartín I, Buerki S, Catalan P. 2013.** Mediterranean origin and Miocene-Holocene Old World diversification of meadow fescues and ryegrasses (*Festuca* subgenus *Schedonorus* and *Lolium*). *Journal of Biogeography* doi:10.1111/jbi.12211.

**Kellogg EA. 2015.** Poaceae. In Kubtizki K, ed., *The Families and Genera of Vascular Plants*. Berlin: Springer.

**Minaya M, Díaz-Pérez A, Mason-Gamer R, Pimentel M, Catalán P. 2015.** Evolution of the beta-amylase gene in the temperate grasses: Non-purifying selection, recombination, semiparalogy, homeology and phylogenetic signal. *Molecular Phylogenetics and Evolution* **91:** 68–85.

**Petersen G, Seberg O, Yde M, Berthelsen K. 2006.** Phylogenetic relationships of Triticum and *Aegilops* and evidence for the origin of the A, B, and D genomes of common wheat (*Triticum aestivum*). *Molecular Phylogenetics and Evolution* **39:** 70–82.

**Pimentel M, Sahuquillo E, Torrecilla Z, Popp M, Catalán P, Brochmann C. 2013.** Hybridization and long-distance colonization at different time scales: towards resolution of long-term controversies in the sweet vernal grasses (*Anthoxanthum*). *Annals of Botany* **112:** 1015-1030.

**Quintanar A, Castroviejo S, Catalán P. 2007.** Phylogeny of the tribe Aveneae (Pooidea, Poaceae) inferred from plastid *TrnT-F* and nuclear ITS sequences. *American Journal of Botany* **94:** 1554–1569.

**Quintanar A, Castroviejo S, Catalán P. 2010.** *A Review of the Systematics and Phylogenetics of the Koeleriinae (Poaceae: Poeae).* In: Seberg O, Petersen G, Barfod AS, Davis J, eds. *Diversity, Phylogeny and Evolution in the Monocotyledons*. Copenhagen: Aarhus University Press, 539-556.

**Saarela JM, Liu Q, Peterson PM, Soreng RJ, Paszko B. 2010.** Phylogenetics of the grass ‘Aveneae-type plastid DNA clade’ (Poaceae: Pooideae, Poeae) based on plastid and nuclear ribosomal DNA sequence data. In: Seberg O, Petersen G, Barfod AS, Davis J, eds. *Diversity, Phylogeny, and Evolution in the Monocotyledons*. Copenhagen: Aarhus University Press, 557–587.

**Saarela JM, Wysocki WP, Barrett CF, Soreng RJ, Davis JI, Clark LG, Kelchner SA, Pires JC, Edger PP, Mayfield DR, Duvall MR. 2015.** Plastid phylogenomics of the cool-season grass subfamily: Clarification of relationships among early-diverging tribes. *AoB PLANTS* **7:** 1–27.

**Schneider J, Winterfeld G, Hoffmann MH, Röser M. 2011.** Duthieeae, a new tribe of grasses (Poaceae) identified among the early diverging lineages of subfamily Pooideae: molecular phylogenetics, morphological delineation, cytogenetics, and biogeography**.** *Systematics and Biodiversity* **9:** 27–44.

**Schneider J, Winterfeld G, Röser M. 2012.** Polyphyly of the grass tribe Hainardieae (Poaceae: Pooideae): identification of its different lineages based on molecular phylogenetics, including morphological and cytogenetic characteristics. *Organism Diversity and Evoltion* **12:**113–132.

**Soreng RJ, Davis JI, Monica A, Voionmaa MA. 2007.** A Phylogenetic Analysis of Poaceae Tribe Poeae *sensu lato* based on morphological characters and sequence data from three plastid-encoded genes: evidence for reticulation, and a new classification for the Tribe. *Kew Bulletin* **62:** 425-454.

**Soreng RJ, Bull RD, Gillespie LJ. 2010.** Phylogeny and reticulation in *Poa* L. based on plastid trnTLF and nrITS sequences with attention to diploids. In: Seberg O, Petersen G, Barfod AS, Davis JI, eds. *Diversity, Phylogeny and Evolution in the Monocotyledons*. Aarhus: Aarhus University Press, 619-643.

**Soreng RJ, Peterson PM, Romaschenko K, Davidse G, Zuloaga FO, Judziewicz EJ, Filgueiras TS, Davis JI, Morrone O. 2015.** A worldwide phylogenetic classification of the Poaceae (Gramineae). *Journal of Systematics and Evolution* **53:** 117-137.

**Voshell SM, Baldini RM, Kumar R, Tatalovich N, Hilu KW. 2011.** Canary grasses (*Phalaris*, Poaceae): Molecular phylogenetics, polyploidy and floret evolution. *Taxon* **60 (5):** 1306–1316.

**Watson L, Dallwitz MJ. 1992** **onwards**. *The grass genera of the world: descriptions, illustrations, identification, and information retrieval; including synonyms, morphology, anatomy, physiology, phytochemistry, cytology, classification, pathogens, world and local distribution, and references*. Version: 18th December 2012.

**Winterfeld G, Perner K, Röser M. 2011.** Genome composition and origin of the polyploid Aegean grass *Avenula agropyroides* (Poaceae). *Journal of Biogeography* **38:** 727-741.

**Winterfeld G, Schneider J, Perner K, Röser M. 2014.** Polyploidy and hybridization as main factors in speciation: complex reticulate evolution in the grass genus *Helictochloa*. *Cytogenetic and Genome Research* **142:** 204-225.
