# Supplementary material for: Are diversification rates and chromosome evolution in the temperate grasses (Pooideae) associated with major environmental changes in the Oligocene-Miocene?
Source: PeerJ. 2017 Sep 22;5:e3815. doi: 10.7717/peerj.3815 (PMC5611942; doi:10.7717/peerj.3815)
Supplement: Table S3 — Models tested, parameters included and AIC values obtained per model in the chromosome base number evolution analysis conducted using CHROMEVOL 2.0 [for a full description of the models and parameters see Mayrose (2014)]. G(ConstR), rate for ascending dysploidy (single chromosome gain); L(ConstR), rate for descending dysploidy (single chromosome loss); BN(R), rate for transitions by base number; BN, base number, a specified chromosome number that characterizes a phylogenetic group; D(ConstR), rate for whole genome duplication; DP(R), rate for demi-duplication (a multiplication of the chromosome number by a factor of 1.5); G(LinearR), rate for ascending dysploidy dependent on the current chromosome number; L(LinearR), rate for descending dysploidy dependent on the current chromosome number. [file peerj-05-3815-s005.doc]

**Table S3.** Models tested, parameters included and AIC values obtained *per* model in the chromosome base number evolution analysis conducted using CHROMEVOL 2.0 [for a full description of the models and parameters see Mayrose (2014)]. G(ConstR), rate for ascending dysploidy (single chromosome gain); L(ConstR), rate for descending dysploidy (single chromosome loss); BN(R), rate for transitions by base number; BN, base number, a specified chromosome number that characterizes a phylogenetic group; D(ConstR), rate for whole genome duplication; DP(R), rate for demi-duplication (a multiplication of the chromosome number by a factor of 1.5); G(LinearR), rate for ascending dysploidy dependent on the current chromosome number; L(LinearR), rate for descending dysploidy dependent on the current chromosome number.

| **Model** | **Parameters** | **AIC value** |
| --- | --- | --- |
| *Base_number* | G(ConstR); L(ConstR); BN(R); BN | 92.294 |
| *Base_number_dupl* | G(ConstR); L(ConstR); D(ConstR); BN(R); BN | 94.295 |
| *Const_rate* | G(ConstR), L(ConstR), D(ConstR) | 97.301 |
| *Const_rate_demi* | G(ConstR); L(ConstR); [D(ConstR) = DP(R)] | 98.656 |
| *Const_rate_demi_est* | G(ConstR); L(ConstR); D(ConstR); DP(R) | 99.301 |
| *Linear_rate* | G(ConstR); G(LinearR); L(ConstR); L(LinearR); D(ConstR) | 101.379 |
| *Linear_rate_demi* | G(ConstR); G(LinearR), L(ConstR), L(LinearR),_[D(ConstR) = DP(R)] | 102.735 |
| *Linear_rate_demi_est* | G(ConstR), G(LinearR), L(ConstR), L(LinearR), D(ConstR), DP(R) | 103.380 |
| *Linear_rate_no_dupli* | G(ConstR), G(LinearR), L(ConstR), L(LinearR) | 142.416 |
| *Const_rate_no_dupli* | G(ConstR), L(ConstR) | 144.017 |
